# *TP73 G4C14-A4T14* polymorphism and cancer susceptibility: evidence from 36 case–control studies

**DOI:** 10.1042/BSR20181452

**Published:** 2018-12-14

**Authors:** Jialin Meng, Shuo Wang, Meng Zhang, Song Fan, Li Zhang, Chaozhao Liang

**Affiliations:** 1 Department of Urology, The First Affiliated Hospital of Anhui Medical University, Anhui Medical University, Hefei, Anhui, China; 2 Institute of Urology, Anhui Medical University, Hefei, Anhui, China; 3 Anhui Province Key Laboratory of Genitourinary Diseases, Anhui Medical University, Hefei, Anhui, China; 4 The First Clinical College of Anhui Medical University, Hefei, Anhui, China

**Keywords:** cancer, genetic variation, G4C14-A4T14, meta-analysis, polymorphism, tumor protein P73

## Abstract

*G4C14-A4T14* polymorphism of *TP73* gene has been reported with a potential association in cancer risks through affected cell homeostasis; however the results were not consistent. We performed a comprehensive meta-analysis to explore the associations between *G4C14-A4T14* polymorphism and cancer susceptibility. Extensive retrieve was performed in PubMed, EMBASE, Google Scholar, Web of Science, Wanfang database and CNKI database up to May 20, 2018. Odds ratios (ORs) and 95% confidence intervals (CIs) were conducted to evaluate the overall strength of the associations in five genetic models, as well as in subgroup analyses. *Q*-test, false-positive report probability analysis and trial sequential analysis, Egger’s test and Begg’s funnel plot were applied to evaluate the robustness of the results. *In silico* analysis was managed to demonstrate the relationship of TP73 expression correlated with cancer tissues. Finally, 36 case–control studies with a total of 9493 cancer cases and 13,157 healthy controls were enrolled into the meta-analysis. The pooled results present a significantly higher risk of *G4C14-A4T14* polymorphism in all the five genetic models, as well as in the subgroups of Caucasian, cervical cancer, colorectal cancer, H-B subgroup and comfort to Hardy–Weinberg equilibrium subgroup. *In silico* analysis revealed that the expression of TP73 in cervical cancer tissue is higher than it in corresponding normal tissue, as well as in cervical cancer. All in all, *TP73 G4C14-A4T14* polymorphism causes an upgrade cancer risk, especially in Caucasian population. *G4C14-A4T14* polymorphism might be a potential biomarker for judging the tumorigenesis of cervical cancer and colorectal cancer.

## Introduction

Cancer is a pivotal public health and leads to the second cause of death problem around the world. In 2018, there are almost 4700 new cancer diagnoses per day, as well as about 1700 cancer-related deaths in United States [1]. Breast cancer, lung cancer and colorectal cancer are the most three frequently cancer of female in United States, while prostate cancer occupied the first diagnosis cancer in male [1]. Attributed to the increasing population growth and aging, cancer has also been the leading cause of death around China. In 2015, there are about 12,000 newly diagnosed invasive cancer cases on average per day, while over 7500 cancer death [2]. In the past decades, biological scientists have reported that environmental factors, genetic mutations and the multiple interactions between them mainly affect the process of tumorigenesis, and the new research results are also on the road, such as epigenetic control [3–5].

Tumor protein P73 (TP73), also known as P53-like transcription factor, is a pivotal member of TP53 family, which affects cell proliferation, apoptosis and cell-cycle regulation [6–8]. Compared with frequently mutant *TP53* gene, *TP73* is rarely mutated [9]. p73 protein, the encoded product of *TP73*, is homologous with p53, 63% of p73 has the same amino acid sequence with p53, so it plays a critical role in normal cell homeostasis, while it can partially compensate the loss of p53 protein function [10,11].

*G4A (rs2273953)* and *C14T (rs1801173)*, the two single-nucleotide polymorphisms (SNPs) of *TP73* at positions 4 (G>A) and 14 (C>T), are incomplete linkage disequilibrium with each other, so we called it as *G4C14-A4T14. G4C14-A4T14* is located at the upstream of *TP73* promoter in exon 2, it could influence the expression of *TP73* through a stem–loop structure [12,13]. In recent years, *G4C14-A4T14* polymorphism of *TP73* was identified implicated in the tumorigenesis of a variety of cancer types, including breast cancer, colorectal cancer, lung cancer, cervical cancer, esophageal cancer and so on [14–17]. Nevertheless, data arising from these published case–control studies were not consistent. One single study may have no sufficient power to identify slight influences of these polymorphisms on cancer susceptibility. Therefore, we conducted a comprehensive meta-analysis to explore the association between *G4C14-A4T14* polymorphism and cancer susceptibility.

## Materials and methods

### Literature search and study selection criteria

We conducted a comprehensive literature search from PubMed, EMBASE, Google Scholar, Web of Science, Wanfang database and CNKI database (up to May 20, 2018). The keywords applied to literature retrieve are as follows: “TP73 OR (Tumor Protein P73) OR (P53-Like Transcription Factor)” AND “cancer OR carcinoma OR tumor OR tumor OR neoplasm.” AND “SNP OR mutation OR variant OR polymorphism”. Furthermore, the references from eligible studies were manually checked for additional relevant literature. The titles and abstracts of identifying studies were examined to exclude obvious irrelevant records. The full-text of the remaining articles was further carefully inspected to determine whether to report the correlation of between *G4C14-A4T14* polymorphism and cancer susceptibility.

All the eligible studies should fulfill the following inclusion criteria: (1) case–control studies focus on the correlation between *G4C14-A4T14* polymorphism and cancer susceptibility; (2) genotype frequency of the cases and controls could be obtained directly or indirectly through calculation; and (3) articles in English or Chinese. On the contrary, studies would be removed if they were: (1) case–report, meta-analysis, systematic review or repetitive publication; (2) lack of genotype frequency data; and (3) publications conducted on animals or cell lines.

### Data extraction

Two independent investigators separately extracted the relative data with any disagreement resolved by rechecking and discussion. For every eligible study, the following data were extracted: the name of the first author, the data of publication, ethnicity, sample size, genotyping methods, and genotype frequency of the cases and controls. In the subgroup analysis by race, the Caucasian population typically lived in Europe or America, and the Asian population typically lived in Asia.

### Statistical methods

All the statistical calculation was conducted with STATA 12.0 software (Stata, College Station, Texas) in the present study. ORs with corresponding 95% CIs were performed to measure the strength of the relationship between *G4C14-A4T14* polymorphism and cancer susceptibility. Five common genetic models applied for assessing gene–disease associations are allele contrast model (GC vs. AT), homozygote comparison model (GC/GC vs. AT/AT), heterozygote comparison model (GC/AT vs. AT/AT), dominant comparison model (GC/GC+GC/AT vs. AT/AT) and recessive comparison model (GC/GC vs. GC/AT+AT/AT) (AT/AT, homozygotes for the common allele; GC/AT, heterozygotes; GC/GC, homozygotes for the rare allele). Stratified analyses were also calculated by ethnicity, cancer type and the source of control. In addition, we applied the chi-squared (*χ*^2^)-based *Q*-test to calculate between-study heterogeneity [18]. *P*<0.1 was indicated as a substantial level of heterogeneity, and a random-effects model (the DerSimonian and Laird method) was selected to pool the data [19]; or else, the fixed-effects model (the Mantel–Haenszel method) was adopted. Moreover, we also conducted the Begg’s funnel plots and Egger’s test to evaluate the publication bias [20,21]. Hardy–Weinberg equilibrium (HWE) of controls was calculated by the χ^2^ test to compare the expected and actual genotype frequencies among the controls in each study. All the statistical tests in this meta-analysis were two-tailed, and *P*-values ≤ 0.05 were considered statistically significant.

### False-positive report probability analysis and trial sequential analysis

We also use the false-positive report probability (FPRP) method to evaluate the results. 0.2 was set as an FPRP threshold and assigned a prior probability of 0.1 to detect the odds ratio (OR) of 0.67/1.50 (protective/risk effects). The significant result with the FPRP values less than 0.2 was considered as a worthy finding [22,23]. Trial sequential analysis (TSA) was conducted with the guideline of a former publication. We set a significance of 5% for type I error, as well as a 30% significance of type II error, to calculate the required sample size, and built the TSA monitoring boundaries.

### *In silico* analysis of TP73 expression

In order to further explore the relationship between *TP73* expression and cancer, we used a newly developed interactive web server, GEPIA (http://gepia.cancer-pku.cn/), to see the difference between tumor tissue and normal tissue. GEPIA provided the mRNA sequencing expression data of tumors and normal samples from the TCGA and the GTEx projects [24].

## Results

### Study characteristics

**Figure 1:**
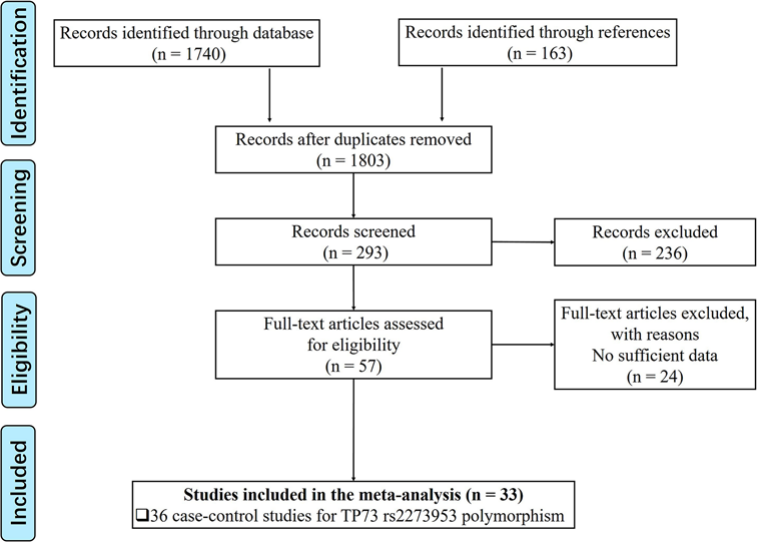
Flowchart presenting the study selection procedure

As shown in Figure 1, we found 1740 potentially relevant studies from PubMed, EMBASE, Google Scholar, Web of Science, Wanfang database and CNKI database. After reviewing titles and abstracts, we excluded 1537 publications not investigating the association between *TP73 G4C14-A4T14* polymorphism and cancer risk. And then, full texts of remaining articles were evaluated. In the end, 36 case–control studies with a total of 9493 cancer cases and 13,157 healthy controls were enrolled into the meta-analysis [14–17,25–53]. The characteristics of these studies were showed in Table 1. Among these publications, there are 6 concerned about cervical cancer [17,34,40,50–52], 5 about lung cancer [16,29,33,35,38], 4 about colorectal cancer [28,37,46,47], 4 about esophageal cancer [14,27,28,39], 4 about gastric cancer [28,39,43,48], 3 about breast cancer [15,26,30], 3 about squamous cell carcinoma of the head and neck [32,41,45], as well as other 7 publications focus on Endometrial cancer [36], lymphoma [31], melanoma [42], nasopharyngeal carcinoma [53], neuroblastoma [25], ovarian cancer [44] and prostate cancer [49], respectively. As to the ethnicity, 14 studies were performed in Caucasians, while the other 22 studies were managed in Asian population. The characteristics of each case–control study, genotype frequencies and HWE examination results were presented in Table 1. Four case–control studies were not comforted to HWE [16,32,37,45], and we further conducted a sensitive analysis to validate the influence of the three studies on the integrated data. In order to evaluate the quality of each enrolled studies, we applied Newcastle–Ottawa Scale (NOS) [45] and fill the result in Table S1, the result of PRISMA2009 checklist was also listed to present our meta-analysis work (Table S2).

**Table 1:** Characteristics of the enrolled studies on *TP73 G4C14-A4T14* polymorphism and cancer

First author	Year	Ethnicity	Genotyping method	Source of control	Cancer type	HWE	Case	Control				
							PAA	PAB	PBB	HAA	HAB	HBB
Romain et al.	1999	Caucasian	PCR	P-B	Neuroblastoma	Y	31	39	3	94	49	7
Ahomadegbe et al*.*	2000	Caucasian	PCR	H-B	Breast cancer	Y	36	22	1	27	7	0
Ryan et al*.*	2001	Caucasian	PCR	P-B	Esophageal cancer	Y	42	41	1	72	65	15
Hamajima et al*.*	2002	Asian	PCR–CTPP	H-B	Esophageal cancer	Y	67	29	6	133	98	10
Hamajima et al*.*	2002	Asian	PCR–CTPP	H-B	Gastric cancer	Y	84	51	9	133	98	10
Hamajima et al*.*	2002	Asian	PCR–CTPP	H-B	Colorectal cancer	Y	87	50	10	133	98	10
Hiraki et al*.*	2003	Asian	PCR–CTPP	H-B	Lung cancer	Y	109	68	12	130	95	10
Huang et al*.*	2003	Asian	PCR–CTPP	P-B	Breast cancer	Y	118	64	18	153	112	17
Hishida et al*.*	2004	Asian	PCR–CTPP	H-B	Lymphoma	Y	49	43	11	261	152	27
Li(a) et al*.*	2004	Caucasian	PCR–CTPP	H-B	SCCHN	N	399	271	38	773	387	69
Li(b) et al*.*	2004	Caucasian	PCR	P-B	Lung cancer	Y	593	394	67	721	365	53
Niwa(a) et al*.*	2004	Asian	PCR–CTPP	H-B	Cervical cancer	Y	57	52	3	270	150	22
Hu et al*.*	2005	Asian	PCR-SSCP	P-B	Lung cancer	Y	255	149	21	295	248	45
Niwa(b) et al*.*	2005	Asian	PCR	H-B	Endometrial cancer	Y	61	39	14	270	150	22
Pfeifer et al*.*	2005	Caucasian	PCR–RFLP	P-B	Colorectal cancer	N	113	54	12	159	96	5
Choi et al*.*	2006	Asian	PCR	P-B	Lung cancer	Y	320	221	41	338	212	32
Ge et al*.*	2006	Asian	PCR–RFLP	H-B	Gastric cancer	Y	146	99	14	391	210	29
Ge et al*.*	2006	Asian	PCR–RFLP	H-B	Esophageal cancer	Y	214	113	21	391	210	29
Zheng et al*.*	2006	Asian	PCR–RFLP	P-B	Cervical cancer	Y	58	22	2	77	19	4
Chen et al*.*	2008	Caucasian	PCR–RFLP	P-B	SCCHN	Y	195	111	20	214	115	20
Li(c) et al*.*	2008	Caucasian	PCR	H-B	Melanoma	Y	468	287	150	497	302	39
Zheng et al.	2008	Asian	PCR–CTPP	P-B	Cervical cancer	Y	71	28	2	77	19	4
Deo Feo et al*.*	2009	Caucasian	PCR	H-B	Gastric cancer	Y	84	22	8	214	71	10
Kang et al*.*	2009	Asian	PCR	P-B	Ovarian cancer	Y	164	74	19	151	92	14
Misra et al*.*	2009	Caucasian	PCR	H-B	SCCHN	N	112	176	15	186	124	9
Lee et al*.*	2010	Asian	PCR–CTPP	P-B	Colorectal cancer	Y	183	171	29	271	173	25
Shirai et al*.*	2010	Asian	PCR–CTPP	H-B	Gastric cancer	Y	220	142	26	239	156	24
Arfaoui et al*.*	2010	Caucasian	PCR	P-B	Colorectal cancer	Y	77	47	26	109	73	22
Mittal et al*.*	2011	Caucasian	PCR–RFLP	P-B	Prostate cancer	Y	121	56	0	192	66	7
Craveiro et al*.*	2012	Caucasian	PCR	P-B	Cervical cancer	Y	95	38	8	119	48	9
Sun et al*.*	2012	Asian	PCR–CTPP	P-B	Cervical cancer	Y	107	100	11	128	80	12
Umar et al*.*	2012	Caucasian	PCR	P-B	Esophageal cancer	Y	174	70	11	200	51	4
Zhou et al*.*	2012	Asian	MALDI-TOF	P-B	Breast cancer	Y	106	59	5	100	67	11
Zhang et al*.*	2014	Asian	PCR	P-B	Nasopharyngeal carcinoma	Y	163	116	14	247	120	13
Wang et al*.*	2014	Asian	PCR–CTPP	P-B	Lung cancer	N	101	59	8	102	68	25
Feng et al.	2017	Asian	PCR	H-B	Cervical cancer	Y	103	67	10	114	55	11

Abbreviations: H-B, hospital based; HWE, Hardy–Weinberg equilibrium; N, polymorphisms did not conform to HWE in the control group; P-B, population based; SCCHN, squamous cell carcinoma of the head and neck; Y, polymorphisms conformed to HWE in the control group.

### Quantitative synthesis

**Figure 2:**
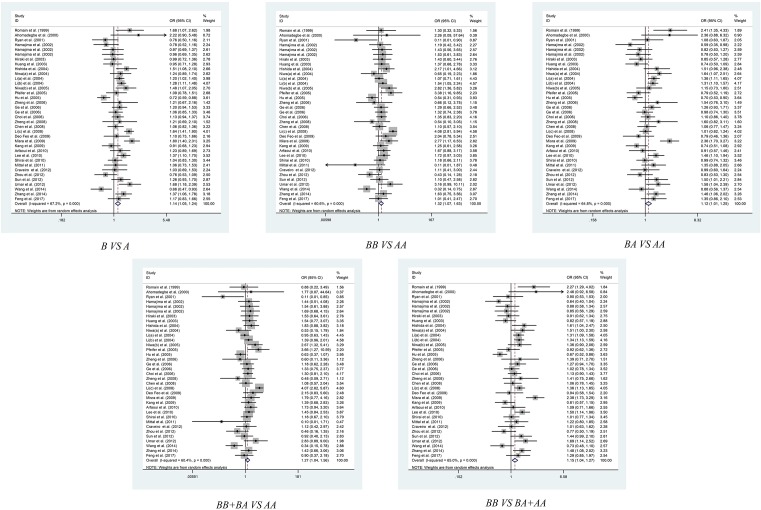
Meta-analysis of the association between *TP73 G4C14-A4T14* polymorphism and overall cancer risk

Table 2 listed the main results of current meta-analysis work of polymorphisms in *G4C14-A4T14* and risk of cancer. The pooled results of the 36 included studies had shown that *G4C14-A4T14* polymorphism conferred a significantly higher overall risk to cancer susceptibility in all the five genetic models, allelic contrast model (GC vs. AT: OR = 1.139, 95% CI = 1.048–1.238, *P*=0.002), homozygote comparison model (GC/GC vs. AT/AT: OR = 1.320, 95% CI = 1.071–1.627, *P*=0.009), heterozygote comparison model (GC/AT vs. AT/AT: OR = 1.123, 95% CI = 1.012–1.245, *P*=0.028), dominant comparison model (GC/GC+GC/AT vs. AT/AT: OR = 1.152, 95% CI = 1.044–1.272, *P*=0.005) and recessive comparison model (GC/GC vs. GC/AT+AT/AT: OR = 1.273, 95% CI = 1.038–1.563, *P*=0.021) (Table 2 and Figure 2).

**Table 2:** Results of pooled analysis for *TP73 G4C14-A4T14* polymorphism and cancer susceptibility

Comparison	Subgroup	*N*	*P*_H_	*P*_Z_	Random	Fixed
B vs. A	Overall	36	<0.001	0.002*	1.139 (1.048–1.238)	1.170 (1.119–1.223)
	Caucasian	14	0.001	<0.001*	1.279 (1.131–1.446)	1.317 (1.232–1.407)
	Asian	22	<0.001	0.228	1.062 (0.963–1.172)	1.060 (0.998–1.126)
	Breast cancer	3	0.091	0.940	0.985 (0.666–1.457)	0.929 (0.747–1.156)
	Colorectal cancer	4	0.339	0.011*	1.197 (1.027–1.395)	1.204 (1.044–1.389)
	SCCHN	3	0.007	0.062	1.308 (0.987–1.733)	1.274 (1.134–1.432)
	Cervical cancer	6	0.982	0.031*	1.190 (1.016–1.393)	1.189 (1.016–1.392)
	Esophageal cancer	4	0.010	0.873	1.027 (0.738–-1.430)	1.057 (0.903–1.236)
	Gastric cancer	4	0.739	0.261	1.084 (0.943–1.247)	1.084 (0.942–1.247)
	Lung cancer	5	<0.001	0.657	0.943 (0.726–1.224)	1.034 (0.945–1.132)
	P-B	20	<0.001	0.176	1.082 (0.965–1.213)	1.098 (1.033–1.168)
	H-B	16	<0.001	0.001*	1.213 (1.079–1.365)	1.256 (1.177–1.340)
	HWE(Y)	32	<0.001	0.003*	1.138 (1.044–1.239)	1.165 (1.109–1.222)
	HWE(N)	4	<0.001	0.484	1.132 (0.799–1.604)	1.200 (1.071–1.345)
BB vs. AA	Overall	36	<0.001	0.009*	1.320 (1.071–1.627)	1.420 (1.265–1.593)
	Caucasian	14	<0.001	0.011*	1.649 (1.119–2.431)	1.806 (1.523–2.142)
	Asian	22	0.033	0.151	1.170 (0.944–1.450)	1.152 (0.984–1.350)
	Breast cancer	3	0.189	0.952	0.918 (0.370–2.279)	0.983 (0.558–1.732)
	Colorectal cancer	4	0.676	0.001*	1.807 (1.258–2.595)	1.820 (1.270–2.608)
	SCCHN	3	0.136	0.196	1.336 (0.807–2.211)	1.235 (0.897–1.699)
	Cervical cancer	6	0.949	0.697	0.925 (0.590–1.451)	0.916 (0.587–1.428)
	Esophageal cancer	4	0.048	0.734	1.165 (0.484–2.804)	1.168 (0.762–1.79)
	Gastric cancer	4	0.815	0.114	1.351 (0.935–1.951)	1.345 (0.931–1.944)
	Lung cancer	5	0.001	0.748	0.912 (0.522–1.595)	1.039 (0.823–1.311)
	P-B	20	0.002	0.470	1.107 (0.841–1.457)	1.136 (0.968–1.335)
	H-B	16	0.001	0.001*	1.625 (1.210–2.183)	1.809 (1.532–2.136)
	HWE(Y)	32	<0.001	0.007*	1.342 (1.085–1.659)	1.476 (1.303–1.671)
	HWE(N)	4	0.001	0.579	1.288 (0.526–3.152)	1.117 (0.819–1.524)
BA vs. AA	Overall	36	<0.001	0.028*	1.123 (1.012–1.245)	1.133 (1.070–1.200)
	Caucasian	14	<0.001	0.008*	1.252 (1.061–1.477)	1.251 (1.149–1.362)
	Asian	22	<0.001	0.458	1.049 (0.924–1.191)	1.044 (0.966–1.129)
	Breast cancer	3	0.100	0.284	0.941 (0.587–1.510)	0.859 (0.651–1.134)
	Colorectal cancer	4	0.026	0.901	0.978 (0.693–1.381)	1.059 (0.879–1.276)
	SCCHN	3	0.002	0.051	1.494 (0.998–2.236)	1.446 (1.246–1.678)
	Cervical cancer	6	0.748	0.001*	1.414 (1.159–1.725)	1.413 (1.159–1.722)
	Esophageal cancer	4	0.031	0.953	1.011 (0.702–1.457)	1.023 (0.841–1.244)
	Gastric cancer	4	0.295	0.867	1.007 (0.821–1.234)	1.015 (0.849–1.214)
	Lung cancer	5	0.002	0.781	0.964 (0.742–1.251)	1.044 (0.929–1.172)
	P-B	20	<0.001	0.129	1.113 (0.969–1.279)	1.118 (1.033–1.209)
	H-B	16	<0.001	0.129	1.134 (0.964–1.334)	1.152 (1.059–1.253)
	HWE(Y)	32	<0.001	0.065	1.101 (0.994–1.219)	1.098 (1.032–1.169)
	HWE(N)	4	<0.001	0.325	1.245 (0.805–1.927)	1.348 (1.165–1.560)
BB+BA vs. AA	Overall	36	<0.001	0.005*	1.152 (1.044–1.272)	1.174 (1.111–1.240)
	Caucasian	14	0.004	<0.001*	1.312 (1.140–1.511)	1.327 (1.224–1.440)
	Asian	22	<0.001	0.332	1.063 (0.940–1.203)	1.059 (0.984–1.141)
	Breast cancer	3	0.096	0.860	0.959 (0.605–1.520)	0.880 (0.675–1.148)
	Colorectal cancer	4	0.073	0.538	1.092 (0.824–1.447)	1.151 (0.964–1.375)
	SCCHN	3	0.001	0.061	1.482 (0.982–2.237)	1.415 (1.226–1.633)
	Cervical cancer	6	0.861	0.003*	1.339 (1.107–1.619)	1.338 (1.106–1.618)
	Esophageal cancer	4	0.020	0.910	1.022 (0.706–1.477)	1.050 (0.870–1.267)
	Gastric cancer	4	0.477	0.522	1.058 (0.892–1.255)	1.057 (0.892–1.254)
	Lung cancer	5	<0.001	0.694	0.943 (0.704–1.263)	1.045 (0.935–1.167)
	P-B	20	<0.001	0.130	1.112 (0.969–1.275)	1.124 (1.043–1.211)
	H-B	16	<0.001	0.012*	1.204 (1.042–1.392)	1.234 (1.139–1.337)
	HWE(Y)	32	<0.001	0.010*	1.138 (1.032–1.255)	1.150 (1.084–1.221)
	HWE(N)	4	<0.001	0.376	1.223 (0.783–1.911)	1.315 (1.143–1.512)
BB vs. BA+AA	Overall	36	<0.001	0.021*	1.273 (1.038–1.563)	1.374 (1.227–1.538)
	Caucasian	14	<0.001	0.046*	1.509 (1.008–2.261)	1.697 (1.437–2.005)
	Asian	22	0.074	0.097	1.160 (0.951–1.415)	1.141 (0.976–1.332)
	Breast cancer	3	0.172	0.798	0.984 (0.388–2.493)	1.075 (0.617–1.875)
	Colorectal cancer	4	0.512	0.002*	1.746 (1.228–2.484)	1.760 (1.241–2.496)
	SCCHN	3	0.416	0.642	1.075 (0.782–1.477)	1.078 (0.786–1.477)
	Cervical cancer	6	0.913	0.349	0.825 (0.530–1.286)	0.811 (0.524–1.256)
	Esophageal cancer	4	0.052	0.688	1.193 (0.504–2.824)	1.165 (0.764–1.777)
	Gastric cancer	4	0.717	0.118	1.342 (0.934–1.927)	1.334 (0.929–1.915)
	Lung cancer	5	0.006	0.794	0.938 (0.578–1.521)	1.029 (0.818–1.294)
	P-B	20	0.006	0.532	1.086 (0.839–1.404)	1.106 (0.945–1.296)
	H-B	16	<0.001	0.005*	1.545 (1.139–2.094)	1.734 (1.474–2.039)
	HWE(Y)	32	<0.001	0.011*	1.309 (1.063–1.613)	1.446 (1.280–1.633)
	HWE(N)	4	0.003	0.743	1.145 (0.509–2.579)	1.000 (0.736–1.358)

*P*_H_: *P* value of *Q*-test for heterogeneity test; *P*_Z_: means statistically significant (*P*<0.05); HWE, Hardy–Weinberg equilibrium; N, polymorphisms did not conform to HWE in the control group; P-B, population based; SCCHN, squamous cell carcinoma of the head and neck; Y, polymorphisms conformed to HWE in the control group; *P value less than 0.05 was considered as statistically significant.

### Stratification analysis by cancer type

**Figure 3:**
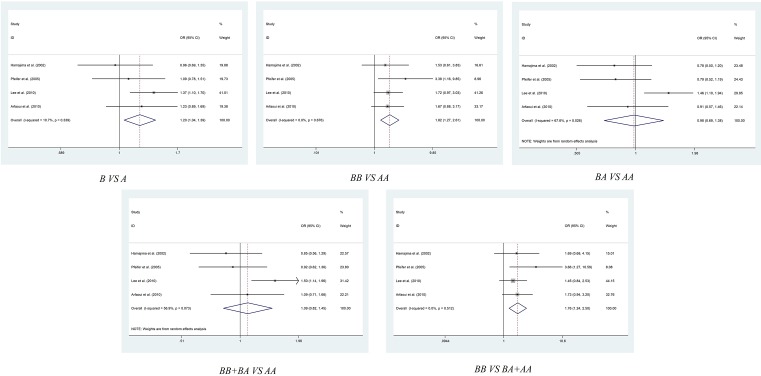
Meta-analysis of the association between *TP73 G4C14-A4T14* polymorphism and colorectal cancer risk

**Figure 4:**
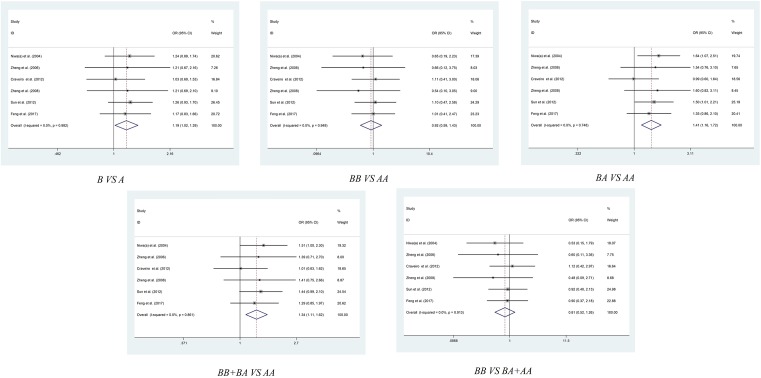
Meta-analysis of the association between *TP73 G4C14-A4T14* polymorphism and cervical cancer risk

After overall pooled analysis, we also conducted stratification analysis by cancer type, in order to obtain more precise result about the *G4C14-A4T14* polymorphism and cancer susceptibility. As shown in Table 2 and Figure 3, the subgroup analysis of six enrolled colorectal cancer related studies have shown that *G4C14-A4T14* polymorphism was related to an increased cancer risk in allelic contrast model (GC vs. AT: OR = 1.204, 95% CI = 1.044–1.389, *P*=0.011), homozygote comparison model (GC/GC vs. AT/AT: OR = 1.820, 95% CI = 1.270–2.608, *P*=0.001) and recessive comparison model (GC/GC vs. GC/AT+AT/AT: OR = 1.760, 95% CI = 1.241–2.496, *P*=0.002). As to cervical cancer, there are also some interesting results. The meta-analysis revealed an increasing risk of cancer caused by *G4C14-A4T14* polymorphism in allelic contrast model (GC vs. AT: OR = 1.189, 95% CI = 1.016–1.392, *P*=0.031), heterozygote comparison model (GC/AT vs. AT/AT: OR = 1.413, 95% CI = 1.159–1.722, *P*=0.001) and dominant comparison model (GC/GC+GC/AT vs. AT/AT: OR = 1.338, 95% CI = 1.106–1.618, *P*=0.003) (Table 2, Figure 4). We also performed subgroup analysis of breast cancer, esophageal cancer, gastric cancer, lung cancer and squamous cell carcinoma of the head and neck, no significant association was found between *G4C14-A4T14* polymorphism and these carcinomas in all five genetic models (Table 2 and Figures S1–S4).

### Stratification analysis by ethnicity

There was some significant result shown in subgroup analysis of ethnicity. The 14 Caucasian based case–control studies shown a significantly increasing risk between *G4C14-A4T14* polymorphism and cancer in allelic contrast model (GC vs. AT: OR = 1.279, 95% CI = 1.131–1.446, *P*<0.001), homozygote comparison model (GC/GC vs. AT/AT: OR = 1.649, 95% CI = 1.119–2.431, *P*<0.001), heterozygote comparison model (GC/AT vs. AT/AT: OR = 1.252, 95% CI = 1.061–1.477, *P*<0.001), dominant comparison model (GC/GC+GC/AT vs. AT/AT: OR = 1.312, 95% CI = 1.140–1.511, *P*=0.004) and recessive comparison model (GC/GC vs. GC/AT+AT/AT: OR = 1.509, 95% CI = 1.008–2.261, *P*<0.001) (Table 2 and Figure S5).

### Stratification analysis by source of control

Due to there are 20 case–control studies based on population controls, whereas another 16 studies enrolled hospital-based controls, we performed the stratified analysis by HWE status to obtain more precise results. The remarkable result shown a noticeable upgrade cancer risk of *G4C14-A4T14* polymorphism of the hospital-based control subgroup in allelic contrast model (GC vs. AT: OR = 1.213, 95%CI = 1.079–1.365, *P*=0.001), homozygote comparison model (GC/GC vs. AT/AT: OR = 1.625, 95% CI = 1.210–2.183 *P*=0.001), dominant comparison model (GC/GC+GC/AT vs. AT/AT: OR = 1.204, 95% CI = 1.042–1.392, *P*=0.012) and recessive comparison model (GC/GC vs. GC/AT+AT/AT: OR = 1.545, 95% CI = 1.139–2.094, *P*=0.005), while there was no significant result of the heterozygote comparison model (GC/AT vs. AT/AT: OR = 1.134, 95% CI = 0.964–1.334, *P*=0.129). Nevertheless, there are no significant result revealed in population-based control subgroup in overall cancer (Table 2 and Figure S6).

### Stratification analysis by HWE status

In order to exclude the influence of allele frequency changing, we calculated whether the control group conform to HWE, and conducted the stratification meta-analysis in subgroups of HWE status. As shown in Table 2 and Figure S7, the subgroup that conforms to HWE was uncovered responsible to the remarkable increasing cancer risk of *G4C14-A4T14* polymorphism in allelic contrast model (GC vs. AT: OR = 1.138, 95%CI = 1.044–1.239, *P*=0.003), homozygote comparison model (GC/GC vs. AT/AT: OR = 1.342, 95% CI = 1.085–1.659, *P*=0.007), dominant comparison model (GC/GC+GC/AT vs. AT/AT: OR = 1.138, 95% CI = 1.032–1.255, *P*=0.010) and recessive comparison model (GC/GC vs. GC/AT+AT/AT: OR = 1.309, 95% CI = 1.063–1.613, *P*=0.011), whereas the other four case–control studies that do not conform to HWE did not influence the result in overall cancer (Table 2 and Figure S7).

### Sensitivity analysis and publication bias

**Figure 5:**
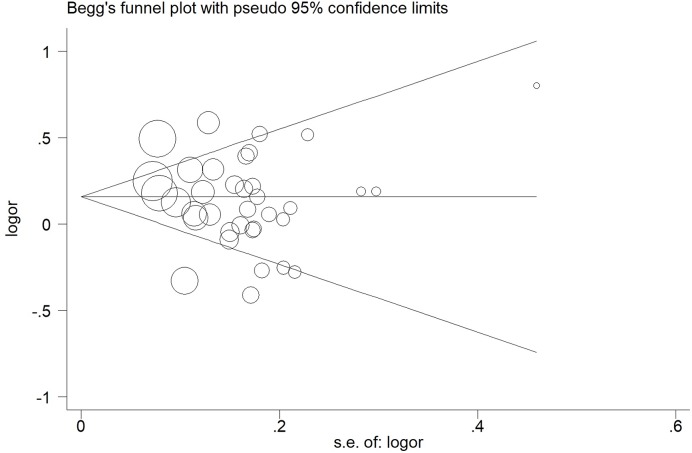
Begg’s funnel plot for publication bias test for *TP73 G4C14-A4T14* polymorphism (GC vs. AT) The *x*-axis is log (OR), and the *y*-axis is natural logarithm of OR. The horizontal line in the figure represents the overall estimated log (OR). The two diagonal lines indicate the pseudo 95% confidence limits of the effect estimate.

Sensitivity analysis was performed to assess the influence of each individual study on the pooled OR by sequential removal of individual studies, the results showed that the study material alteration did not influence the corresponding pooled ORs for the overall meta-analysis (Figure 5 and Table S3). In addition, Begg’s funnel plot and Egger’s test were presented to assess the potential publication bias, no evidence of publication bias was revealed by evaluating the shape of Begg’s funnel plot and by Egger’s regression test (Figures S8, S9 and Table S4).

### Result of FPRP and TSA

**Figure 6:**
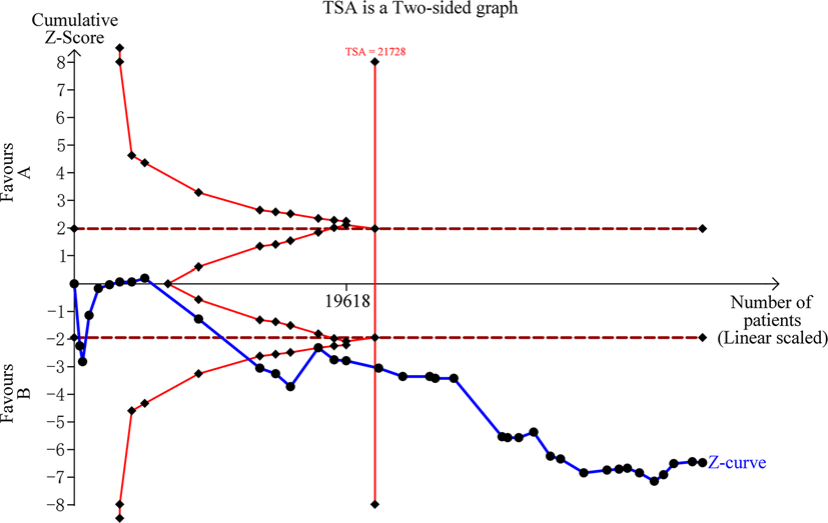
Trial sequential analysis for *TP73 G4C14-A4T14* polymorphism under the allele contrast model

The FPRP values for significant findings at different prior probability levels are shown in Table 3. In the result of overall group in five genetic models, all the statistical power is about 1, and the FPRP values are all less than 0.2, under the prior probability of 0.1. On the subgroup of cervical cancer and colorectal cancer, the FPRP values are also less than 0.2. The result of TSA is shown in Figure 6, the required sample size is 21,728 samples, and the cumulative *z*-curve crossed the trial sequential monitoring boundary before reaching the required sample size, which means that our conclusions are robust with these sufficient evidence.

**Table 3:** False-positive report probability values for associations between the risk of cancer and the frequency of genotypes of TP73 Gene

Comparison	Subgroup	*P*_z_	OR (95% CI)	Statistical power^*^	Prior probability				
					0.250	0.1	0.01	0.001	0.0001
B vs. A	Overall	0.002	1.139 (1.048–1.238)	1.000	<0.001	<0.001	0.001	0.006	0.053
	Caucasian	<0.001	1.279 (1.131–1.446)	0.809	<0.001	<0.001	0.001	0.006	0.054
	Colorectal cancer	0.011	1.204 (1.044–1.389)	0.754	<0.001	<0.001	0.001	0.007	0.062
	Cervical cancer	0.031	1.189 (1.016–1.392)	0.446	<0.001	<0.001	0.001	0.008	0.075
	H-B	0.001	1.213 (1.079–1.365)	1.000	<0.001	<0.001	0.001	0.006	0.054
	HWE(Y)	0.003	1.138 (1.044–1.239)	1.000	<0.001	<0.001	0.001	0.006	0.053
BB vs. AA	Overall	0.009	1.320 (1.071–1.627)	1.000	<0.001	<0.001	0.002	0.024	0.196
	Caucasian	0.011	1.649 (1.119–2.431)	0.467	0.003	0.008	0.081	0.469	0.898
	Colorectal cancer	0.001	1.820 (1.270–2.608)	0.901	0.002	0.005	0.053	0.362	0.850
	H-B	0.001	1.625 (1.210–2.183)	1.000	0.001	0.002	0.017	0.148	0.635
	HWE(Y)	0.007	1.342 (1.085–1.659)	1.000	<0.001	<0.001	0.003	0.025	0.208
BA vs. AA	Overall	0.028	1.123 (1.012–1.245)	0.992	<0.001	<0.001	0.001	0.006	0.053
	Caucasian	0.008	1.252 (1.061–1.477)	0.557	<0.001	<0.001	0.001	0.009	0.085
	Cervical cancer	0.001	1.413 (1.159–1.722)	0.822	<0.001	<0.001	0.002	0.018	0.157
BB+BA vs. AA	Overall	0.005	1.152 (1.044–1.272)	1.000	<0.001	<0.001	0.001	0.006	0.053
	Caucasian	<0.001	1.312 (1.140–1.511)	0.703	<0.001	<0.001	0.001	0.006	0.061
	Cervical cancer	0.003	1.338 (1.106–1.618)	0.714	<0.001	<0.001	0.002	0.015	0.135
	H-B	0.012	1.204 (1.042–1.392)	1.000	<0.001	<0.001	0.001	0.007	0.064
	HWE(Y)	0.010	1.138 (1.032–1.255)	0.996	<0.001	<0.001	0.001	0.006	0.053
BB vs. BA+AA	Overall	0.021	1.273 (1.038–1.563)	1.000	<0.001	<0.001	0.002	0.022	0.182
	Caucasian	0.046	1.509 (1.008–2.261)	0.341	0.003	0.010	0.100	0.528	0.918
	Colorectal cancer	0.002	1.760 (1.241–2.496)	0.888	0.001	0.004	0.045	0.323	0.827
	H-B	0.005	1.545 (1.139–2.094)	1.000	0.001	0.002	0.020	0.172	0.675
	HWE(Y)	0.011	1.309 (1.063–1.613)	1.000	<0.001	<0.001	0.002	0.024	0.195

CI, confidence interval; H-B, hospital based; HWE(Y), Polymorphisms conformed to Hardy–Weinberg equilibrium in the control group; OR, odds ratio.

* Statistical power was calculated using the number of observations in the subgroup and the OR and *P* values in this table.

### *In silico* analysis of TP73 expression

*In silico* analysis, we draw out the correlation between *TP73* expression and breast invasive carcinoma, cervical squamous cell carcinoma and endocervical adenocarcinoma (CESC), colon adenocarcinoma (COAD), esophageal carcinoma, head and neck squamous cell carcinoma, lung adenocarcinoma, lung squamous cell carcinoma (LUSC), ovarian serous, prostate adenocarcinoma, rectum adenocarcinoma, skin cutaneous melanoma, Ssomach adenocarcinoma, with the help of GEPIA web server. The result indicated that the expression of *TP73* in tumor tissue is higher than it in corresponding normal tissue of CESC (TPM = 9.60 vs. 0.58 respectively, *P*<0.01), COAD (TPM = 1.93 vs. 0.56 respectively, *P*<0.01), LUSC (TPM = 7.64 vs. 1.07 respectively, *P*<0.01), whereas lower than it in normal tissue of SKCM (TPM = 0.67 vs. 7.62 respectively, *P*<0.01) ( Figure S10).

## Discussion

*TP73* gene is located at chromosome 1p36 and comprises 15 exons [54]. *TP73* could be transcribed from two individual promoters, one is in the upstream of exon 1, it could produce p53-like proteins containing transactivation domain (TAp73) and another TA lacking protein (ΔTAp73). The second promoter is situated in intron 3, it could turn out the N-terminal truncated isoform (ΔNp73) [55]. What’s more, both TAp73 and ΔNp73 undergo the alternative splicing and initiation of translation, and lead to several splicing isoforms [56,57]. While sharing the similar sequence with p53, TAp73 could activate the expression of downstream genes through specifically binding domain of p53 response element, regulating cell apoptosis or cell-cycle arrest [58,59]. On the meanwhile, ΔNp73 could present a potent anti-oncogenic function through inhibiting the key role of TAp63, TAp73 or p53 [60]. Several publications had reported that the *TP73* expression plays critical role in tumorigenesis, combined with different isoforms or several mutations [61–64].

In the past decades, almost 146 unique variations had been reported (shown in the Biomuta database [65]), while numerous studies had probed into the relationship of *G4C14-A4T14* polymorphism and cancer genomics. *G4A (rs2273953)* and *C14T (rs1801173)* polymorphisms are located at position 4 (G to A) and 14 (C to T) of exon 2 5’-untranslated region, which might influence the initiating AUG codon through constructing a stem–loop [54]. Zheng et al. [40] and Niwa et al. [34] reported that *G4C14-A4T14* polymorphism was not associated with the cancer susceptibility of cervical cancer in Uighur and Japanese, respectively. However, Craveiro et al. [51] revealed that *G4C14-A4T14* polymorphism leads to an increasing risk of cervical cancer, as well as the newest study conducted by Feng et al. [17].As colorectal cancer, Hamajima et al. [28] presented that no significant differences in the genotype frequencies were observed among the enrolled cases and controls in his study. On the contrast, Lee et al. [47] reported that GC/AT and AT/AT genotypes were significantly associated with colorectal cancer risk in Korean population. Arfaoui et al. [66] also uncovered that no remarkable differences of genotype frequencies in cancers and controls, but they found that AT/AT genotype might cause the poor prognosis of colorectal cancer. Several researches also managed in lung cancer. Hu et al. [35] indicated that both AT/AT and GC/AT variants were associated with a remarkable decreased risk for lung cancer, distinguishingly, Li et al. [64] suggested that the AT/AT and GC/AT genotypes were related with a statistically significantly increased risk for lung cancer. Choi et al. [38] did not agree with each of them, they revealed that *TP73 G4C14-A4T14* polymorphism does not affect the susceptibility to lung cancer in Korean population.

Among these publications concerned about *G4C14-A4T14* polymorphism and cancer risk, the result is not consistent. Liang et al. [67] conducted a meta-analysis about G4C14-A4T14 polymorphism and cervical cancer, they only enrolled 5 studies, as well as Liu et al. [68], they only enrolled 5 studies about lung cancer. Yu et al. [69] had performed a meta-analysis with only 23 eligible studies; however, they draw a decreased risk of G4C14-A4T14 polymorphism, this mistake may cause by the fewer samples. Therefore, our team carried out the present comprehensive meta-analysis aiming at shedding light on the multiple lines of evidence. Finally, 36 case–control studies comprise 9493 cases and 13,157 controls were enrolled and analyzed. All in all, our recent updated meta-analysis draws a comprehensive, precise and convincible result, which is that G4C14-A4T14 polymorphism of TP73 is strongly associated with the increasing cancer risk, especially for Caucasian, cervical cancer and colorectal cancer. Therefore, in the future, G4C14-A4T14 polymorphism might be a useful diagnostic marker for cervical cancer and colorectal cancer, especially in Caucasian population. On the other hand, for researchers, other polymorphisms of TP73 should be focused on to assess whether they change cancer risks.

The current result about *G4C14-A4T14* polymorphism and cancer risk should be cautiously interpreted, because there are some limitations. First, an insufficient capacity that slight effects on cancer susceptibility occurred when a stratified analysis was conducted by the cancer type, ethnicity and source of control. Second, several potential confounding factors were ignored, such as age, gender, smoking, drinking and etc., so we are unable to perform a further assessment of potential gene–environment interactions. Third, we only enrolled publications written in English or Chinese, missing publications from other languages may cause potential bias. On the meanwhile, the advantages of this research should not be buried. First, a comprehensive search was conducted to identify more qualified studies, so this analysis is persuasive and substantive. Second, the quality of each registered research was evaluated by NOS scale, low-quality studies were eliminated to raise the credibility of results. Third, stratification analysis was performed by ethnicity, source of controls, tumor type or ethnicity, in order to decrease the impact of heterogeneity and obtain the real conclusion.

In conclusion, our meta-analysis had successfully elaborated that *TP73 G4C14-A4T14* polymorphism causes an upgrade cancer risk, especially in Caucasian population. *G4C14-A4T14* polymorphism might be a potential biomarker for judging the tumorigenesis of cervical cancer and colorectal cancer.

## Supporting information

**Fig. S1:** 

**Fig. S2:** 

**Fig. S3:** 

**Fig. S4:** 

**Fig. S5:** 

**Fig. S6:** 

**Fig. S7:** 

**Fig. S8:** 

**Fig. S9:** 

**Fig. S10:** 

**Supplementary Table 1:** Methodological quality of the included studies according to the Newcastle-Ottawa Scale.

**Supplementary Table 2:** PRISMA 2009 Checklist

**Supplementary Table 3:** Details of the sensitivity analyses for *TP73 G4C14-A4T14* polymorphism and cancer risk.

**Supplementary Table 4:** *P* values of the Egger's test for *TP73 G4C14-A4T14* polymorphism.

## Abbreviations

CESC: cervical squamous cell carcinoma and endocervical adenocarcinoma
COAD: colon adenocarcinoma
HWE: Hardy–Weinberg equilibrium
LUSC: lung squamous cell carcinoma
NOS: Newcastle–Ottawa Scale
SNP: single-nucleotide polymorphism
TSA: trial sequential analysis
TP73: tumor protein P73
