# Supplementary material for: TP73 G4C14-A4T14 polymorphism and cancer susceptibility: evidence from 36 case–control studies
Source: Biosci Rep. 2018 Dec 14;38(6):BSR20181452. doi: 10.1042/BSR20181452 (PMC6294616; doi:10.1042/BSR20181452)
Supplement: Supplementary file 1 [file bsr20181452_Supp1.pdf]

Supplementary tables and figures

Effect of *G4C14-A4T14* Polymorphism on Tumor Protein 73 Gene to Cancer Susceptibility: A Systematic Review and Meta-Analysis.

Jialin Meng[1]; Shuo Wang[2]; Meng Zhang[1]; Song Fan[1]; Li Zhang[1*]; Chaozhao Liang[1*]

1. Department of Urology, The First Affiliated Hospital of Anhui Medical University; Institute of Urology, Anhui Medical University; Anhui Province Key Laboratory of Genitourinary Diseases, Anhui Medical University, Hefei, Anhui, China.

2. The First Clinical College of Anhui Medical University, Hefei, Anhui, China.

Corresponding author:

Chaozhao Liang, MD, PhD, liang_chaozhao@ahmu.edu.cn;

Li Zhang, PhD, lzhang@ahmu.edu.

Running Title: Association between *TP73 G4C14-A4T14* polymorphism and cancer susceptibility

Supplementary table 1. Methodological quality of the included studies according to the Newcastle-Ottawa Scale.

SNP	Author	Year	Adequacy of Case Definition	Representativeness of the Cases	Selection of Controls	Definition of Controls	Comparability Cases/Controls	Ascertainment of Exposure	Same Method of Ascertainment	Non-response rate
rs2273953	Romain *et al.*	1999	*		*	*	**	*	*	NA
rs2273953	Ahomadegbe *et al.*	2000	*			*	*	*	*	NA
rs2273953	Ryan *et al.*	2001	*	*	*	*	**	*	*	NA
rs2273953	Hamajima *et al.*	2002	*	*		*	*	*	*	NA
rs2273953	Hamajima *et al.*	2002	*	*		*	*	*	*	NA
rs2273953	Hamajima *et al.*	2002	*	*		*	*	*	*	NA
rs2273953	Hiraki *et al.*	2003	*	*		*	*	*	*	NA
rs2273953	Huang *et al.*	2003	*	*	*	*	*	*	*	NA
rs2273953	Hishida *et al.*	2004	*	*		*	*	*	*	NA
rs2273953	Li(a) *et al.*	2004	*	*		*	**	*	*	NA
rs2273953	Li(b) *et al.*	2004	*	*		*	**	*	*	NA
rs2273953	Niwa(a) *et al.*	2004	*	*	*	*	*	*	*	NA
rs2273953	Hu *et al.*	2005	*	*		*	**	*	*	NA
rs2273953	Niwa(b) *et al.*	2005	*	*	*	*	*	*	*	NA
rs2273953	Pfeifer *et al.*	2005	*	*	*	*	**	*	*	NA
rs2273953	Choi *et al.*	2006	*	*	*	*	**	*	*	NA
rs2273953	Ge(a) *et al.*	2006	*	*		*	*	*	*	NA
rs2273953	Ge(a) *et al.*	2006	*	*		*	*	*	*	NA
rs2273953	Zheng *et al.*	2006	*	*		*	*	*	*	NA
rs2273953	Chen *et al.*	2008	*	*	*	*	**	*	*	NA
rs2273953	Li(c) *et al.*	2008	*		*	*	**	*	*	NA
rs2273953	Deo Feo *et al.*	2009	*	*		*	*	*	*	NA
rs2273953	Kang *et al.*	2009	*	*		*	**	*	*	NA
rs2273953	Misra *et al.*	2009	*	*		*	*	*	*	NA
rs2273953	Lee *et al.*	2010	*	*	*	*	**	*	*	NA
rs2273953	Shirai *et al.*	2010	*	*	*	*	*	*	*	NA
rs2273953	Arfaoui *et al.*	2010	*	*	*	*	**	*	*	NA
rs2273953	Mittal *et al.*	2011	*	*		*	**	*	*	NA

rs2273953	Craveiro *et al.*	2012	*	*	*	*	**	*	*	NA
rs2273953	Zheng *et al.*	2008	*	*	*	*	*	*	*	NA
rs2273953	Sun *et al.*	2012	*	*	*	*	*	*	*	NA
rs2273953	Umar *et al.*	2012	*	*	*	*	**	*	*	NA
rs2273953	Zhou *et al.*	2012	*	*	*	*	**	*	*	NA
rs2273953	Zhang *et al.*	2014	*	*	*	*	*	*	*	NA
rs2273953	Wang *et al.*	2014	*	*	*	*	**	*	*	NA
rs2273953	Feng *et al.*	2017	*	*		*	**	*	*	NA

This table identifies “high” quality choices with a “star”. A study can be awarded a maximum of 1 star for each numbered item within the Selection and Exposure categories. A maximum of 2 stars can be given for Comparability. *, Yes; NA, not applicable. (http://www.ohri.ca/programs/clinical epidemiology/oxford.htm).

Supplementary table 2 :PRISMA 2009 Checklist

Section/topic	#	Checklist item	Reported on page #
**TITLE**			
Title	1	Identify the report as a systematic review, meta-analysis, or both.	Page 1
**ABSTRACT**			
Structured summary	2	Provide a structured summary including, as applicable: background; objectives; data sources; study eligibility criteria, participants, and interventions; study appraisal and synthesis methods; results; limitations; conclusions and implications of key findings; systematic review registration number.	Page 2
**INTRODUCTION**			
Rationale	3	Describe the rationale for the review in the context of what is already known.	Page 3-4
Objectives	4	Provide an explicit statement of questions being addressed with reference to participants, interventions, comparisons, outcomes, and study design (PICOS).	Page 3-4
**METHODS**			
Protocol and registration	5	Indicate if a review protocol exists, if and where it can be accessed (e.g., Web address), and, if available, provide registration information including registration number.	N/A
Eligibility criteria	6	Specify study characteristics (e.g., PICOS, length of follow-up) and report characteristics (e.g., years considered, language, publication status) used as criteria for eligibility, giving rationale.	Study selection: Page 4
Information sources	7	Describe all information sources (e.g., databases with dates of coverage, contact with study authors to identify additional studies) in the search and date last searched.	Search strategy: Page 4
Search	8	Present full electronic search strategy for at least one database, including any limits used, such that it could be repeated.	Search strategy: Page 4
Study selection	9	State the process for selecting studies (i.e., screening, eligibility, included in systematic review, and, if applicable, included in the meta-analysis).	Figure 1
Data collection process	10	Describe method of data extraction from reports (e.g., piloted forms, independently, in duplicate) and any processes for obtaining and confirming data from investigators.	Data extraction: Page 5
Data items	11	List and define all variables for which data were sought (e.g., PICOS, funding sources) and any assumptions and simplifications made.	Data extraction: Page 5
Risk of bias in individual studies	12	Describe methods used for assessing risk of bias of individual studies (including specification of whether this was done at the study or outcome level), and how this information is to be used in any data synthesis.	Statistical analysis: Page5-6
Summary measures	13	State the principal summary measures (e.g., risk ratio, difference in means).	Statistical analysis: Page5-6
Synthesis of results	14	Describe the methods of handling data and combining results of studies, if done, including measures of consistency (e.g., I^2) for each meta-analysis.	Statistical analysis: Page5-6

Supplementary table 2 :PRISMA 2009 Checklist

Section/topic	#	Checklist item	Reported on page #
Risk of bias across studies	15	Specify any assessment of risk of bias that may affect the cumulative evidence (e.g., publication bias, selective reporting within studies).	Statistical analysis: Page5-6
Additional analyses	16	Describe methods of additional analyses (e.g., sensitivity or subgroup analyses, meta-regression), if done, indicating which were pre-specified.	Statistical analysis: Page5-6
RESULTS			
Study selection	17	Give numbers of studies screened, assessed for eligibility, and included in the review, with reasons for exclusions at each stage, ideally with a flow diagram.	Description of studies: page 6-7
Study characteristics	18	For each study, present characteristics for which data were extracted (e.g., study size, PICOS, follow-up period) and provide the citations.	Table 1-2
Risk of bias within studies	19	Present data on risk of bias of each study and, if available, any outcome level assessment (see item 12).	Page 7-9
Results of individual studies	20	For all outcomes considered (benefits or harms), present, for each study: (a) simple summary data for each intervention group (b) effect estimates and confidence intervals, ideally with a forest plot.	Page 7-9
Synthesis of results	21	Present results of each meta-analysis done, including confidence intervals and measures of consistency.	Page 7-9
Risk of bias across studies	22	Present results of any assessment of risk of bias across studies (see Item 15).	Page 10
Additional analysis	23	Give results of additional analyses, if done (e.g., sensitivity or subgroup analyses, meta-regression [see Item 16]).	Page 10
DISCUSSION			
Summary of evidence	24	Summarize the main findings including the strength of evidence for each main outcome; consider their relevance to key groups (e.g., healthcare providers, users, and policy makers).	Page 10-12
Limitations	25	Discuss limitations at study and outcome level (e.g., risk of bias), and at review-level (e.g., incomplete retrieval of identified research, reporting bias).	Page 12
Conclusions	26	Provide a general interpretation of the results in the context of other evidence, and implications for future research.	Page 12
FUNDING			
Funding	27	Describe sources of funding for the systematic review and other support (e.g., supply of data); role of funders for the systematic review.	Page 13

From: Moher D, Liberati A, Tetzlaff J, Altman DG, The PRISMA Group (2009). Preferred Reporting Items for Systematic Reviews and Meta-Analyses: The PRISMA Statement. PLoS Med 6(7): e1000097. doi:10.1371/journal.pmed1000097

For more information, visit: www.prisma-statement.org.

Supplementary table 3. Details of the sensitivity analyses for *TP73 G4C14-A4T14* polymorphism and cancer risk.

Comparison	Study omitted	Estimate	95% Confident Interval	Effect Model
B VS. A	Romain *et al.* (1999)	1.130111	(1.0389659-1.2292511)	Random
	Ahomadegbe *et al.* (2000)	1.133441	(1.0426028-1.2321934)	
	Ryan *et al.* (2001)	1.149084	(1.0569933-1.2491991)	
	Hamajima *et al.* (2002)	1.149048	(1.0568505-1.2492886)	
	Hamajima *et al.* (2002)	1.143688	(1.0504015-1.2452592)	
	Hamajima *et al.* (2002)	1.143968	(1.0506956-1.2455198)	
	Hiraki *et al.* (2003)	1.143300	(1.049834-1.2450864)	
	Huang *et al.* (2003)	1.144951	(1.051527-1.2466749)	
	Hishida *et al.* (2004)	1.130186	(1.0383892-1.2300983)	
	Li(a) *et al.* (2004)	1.136100	(1.0429964-1.2375143)	
	Li(b) *et al.* (2004)	1.136270	(1.0404902-1.2408674)	
	Niwa(a) *et al.* (2004)	1.132942	(1.0376155-1.2370261)	
	Hu *et al.* (2005)	1.130616	(1.0386425-1.2307329)	
	Niwa(b) *et al.* (2005)	1.140216	(1.0467455-1.2420339)	
	Pfeifer *et al.* (2005)	1.161382	(1.0749881-1.2547184)	
	Choi *et al.* (2006)	1.137864	(1.0454811-1.2384103)	
	Ge *et al.* (2006)	1.136584	(1.0425612-1.2390856)	
	Ge *et al.* (2006)	1.141614	(1.0472397-1.2444935)	
	Zheng *et al.* (2006)	1.138859	(1.0438092-1.2425637)	
	Chen *et al.* (2008)	1.137807	(1.0453632-1.2384263)	
	Li(c) *et al.* (2008)	1.141616	(1.0475813-1.2440913)	
	Zheng *et al.* (2008)	1.121979	(1.036285-1.2147598)	
	Deo Feo *et al.* (2009)	1.139758	(1.0467359-1.2410459)	
	Kang *et al.* (2009)	1.121993	(1.0340479-1.2174183)	
	Misra *et al.* (2009)	1.146506	(1.0533116-1.247947)	
	Lee *et al.* (2010)	1.136432	(1.0431769-1.2380246)	
	Shirai *et al.* (2010)	1.131226	(1.0381091-1.2326947)	
	Arfaoui *et al.* (2010)	1.142575	(1.0483176-1.2453082)	
	Mittal *et al.* (2011)	1.140907	(1.0476701-1.2424422)	

	Craveiro *et al.* (2012)	1.141550	(1.0484502-1.2429176)	
	Sun *et al.* (2012)	1.150812	(1.0588705-1.2507372)	
	Umar *et al.* (2012)	1.135496	(1.0422311-1.2371075)	
	Zhou *et al.* (2012)	1.127594	(1.0368782-1.2262458)	
	Zhang *et al.* (2014)	1.156271	(1.0662039-1.2539471)	
	Wang *et al.* (2014)	1.131914	(1.0390542-1.2330716)	
	Feng *et al.* (2017)	1.137936	(1.0446957-1.2394984)	
	Combined	1.138912	(1.0476518-1.2381208)	
BB vs. AA	Romain *et al.* (1999)	1.319278	(1.0667233-1.6316261)	Random
	Ahomadegbe *et al.* (2000)	1.316493	(1.0663194-1.6253613)	
	Ryan *et al.* (2001)	1.351274	(1.1013309-1.6579402)	
	Hamajima *et al.* (2002)	1.321924	(1.0677318-1.6366303)	
	Hamajima *et al.* (2002)	1.315825	(1.0620592-1.6302253)	
	Hamajima *et al.* (2002)	1.313211	(1.0598292-1.6271713)	
	Hiraki *et al.* (2003)	1.315345	(1.0612687-1.6302501)	
	Huang *et al.* (2003)	1.316119	(1.060487-1.6333713)	
	Hishida *et al.* (2004)	1.298030	(1.047896-1.6078711)	
	Li(a) *et al.* (2004)	1.338100	(1.0834709-1.6525694)	
	Li(b) *et al.* (2004)	1.329958	(1.0691391-1.6544045)	
	Niwa(a) *et al.* (2004)	1.305424	(1.0454627-1.6300255)	
	Hu *et al.* (2005)	1.287425	(1.0418331-1.5909095)	
	Niwa(b) *et al.* (2005)	1.292725	(1.0471598-1.5958755)	
	Pfeifer *et al.* (2005)	1.379596	(1.1278074-1.6875975)	
	Choi *et al.* (2006)	1.330259	(1.0772062-1.6427583)	
	Ge *et al.* (2006)	1.318780	(1.0623056-1.6371746)	
	Ge *et al.* (2006)	1.317213	(1.0599747-1.636878)	
	Zheng *et al.* (2006)	1.315040	(1.0564038-1.6369984)	
	Chen *et al.* (2008)	1.333760	(1.0806208-1.6461972)	
	Li(c) *et al.* (2008)	1.327111	(1.06957-1.6466649)	
	Zheng *et al.* (2008)	1.263788	(1.0609929-1.5053436)	
	Deo Feo *et al.* (2009)	1.304227	(1.0534731-1.6146679)	
	Kang *et al.* (2009)	1.292150	(1.0452878-1.5973122)	
	Misra *et al.* (2009)	1.320516	(1.0643915-1.638271)	

	Lee *et al.* (2010)	1.306278	(1.0520783-1.6218961)	
	Shirai *et al.* (2010)	1.303745	(1.0492596-1.6199538)	
	Arfaoui *et al.* (2010)	1.323549	(1.0655433-1.6440278)	
	Mittal *et al.* (2011)	1.337680	(1.0873282-1.6456749)	
	Craveiro *et al.* (2012)	1.324417	(1.0695929-1.6399503)	
	Sun *et al.* (2012)	1.355713	(1.1013395-1.6688389)	
	Umar *et al.* (2012)	1.325778	(1.0700411-1.6426341)	
	Zhou *et al.* (2012)	1.296641	(1.0498366-1.6014660)	
	Zhang *et al.* (2014)	1.384560	(1.1341154-1.6903105)	
	Wang *et al.* (2014)	1.309388	(1.0558735-1.6237718)	
	Feng *et al.* (2017)	1.328905	(1.0731231-1.6456532)	
	Combined	1.320050	(1.0708865-1.6271875)	
BA vs. AA	Romain *et al.* (1999)	1.106631	(0.9993051-1.2254829)	Random
	Ahomadegbe *et al.* (2000)	1.115426	(1.0057140-1.2371070)	
	Ryan *et al.* (2001)	1.123842	(1.0113208-1.2488818)	
	Hamajima *et al.* (2002)	1.139215	(1.0284778-1.2618757)	
	Hamajima *et al.* (2002)	1.132178	(1.0193869-1.2574497)	
	Hamajima *et al.* (2002)	1.133800	(1.0212653-1.2587351)	
	Hiraki *et al.* (2003)	1.131726	(1.0186899-1.2573041)	
	Huang *et al.* (2003)	1.136576	(1.0244304-1.2609981)	
	Hishida *et al.* (2004)	1.114550	(1.0033197-1.2381121)	
	Li(a) *et al.* (2004)	1.111378	(1.0010409-1.2338771)	
	Li(b) *et al.* (2004)	1.114141	(1.0007142-1.2404241)	
	Niwa(a) *et al.* (2004)	1.115610	(1.0010546-1.2432748)	
	Hu *et al.* (2005)	1.122248	(1.0094269-1.2476787)	
	Niwa(b) *et al.* (2005)	1.133906	(1.0212670-1.2589688)	
	Pfeifer *et al.* (2005)	1.143752	(1.0347936-1.2641829)	
	Choi *et al.* (2006)	1.117569	(1.0064924-1.2409034)	
	Ge *et al.* (2006)	1.118427	(1.0050888-1.2445460)	
	Ge *et al.* (2006)	1.128405	(1.0140483-1.2556573)	
	Zheng *et al.* (2006)	1.123902	(1.0088218-1.2521090)	
	Chen *et al.* (2008)	1.116457	(1.0055597-1.2395855)	
	Li(c) *et al.* (2008)	1.125180	(1.0112145-1.2519893)	

	Zheng *et al.* (2008)	1.127993	(1.0122669-1.2569482)	
	Deo Feo *et al.* (2009)	1.131305	(1.01891540-1.256092)	
	Kang *et al.* (2009)	1.094806	(0.9453852-1.2051811)	
	Misra *et al.* (2009)	1.136959	(1.0248612-1.2613169)	
	Lee *et al.* (2010)	1.128753	(1.0158259-1.2542344)	
	Shirai *et al.* (2010)	1.112129	(1.0005869-1.2361055)	
	Arfaoui *et al.* (2010)	1.128081	(1.0138044-1.2552380)	
	Mittal *et al.* (2011)	1.117390	(1.0052334-1.2420594)	
	Craveiro *et al.* (2012)	1.126159	(1.0133621-1.2515121)	
	Sun *et al.* (2012)	1.131763	(1.0189650-1.2570463)	
	Umar *et al.* (2012)	1.113547	(1.0022472-1.2372072)	
	Zhou *et al.* (2012)	1.112337	(1.0015877-1.2353322)	
	Zhang *et al.* (2014)	1.130211	(1.0172521-1.2557129)	
	Wang *et al.* (2014)	1.112925	(1.0013776-1.2368977)	
	Feng *et al.* (2017)	1.117607	(1.0055333-1.2421712)	
	Combined	1.122900	(1.0124720-1.2453718)	
BB+BA vs. AA	Romain *et al.* (1999)	1.137691	(1.0313668-1.2549757)	Random
	Ahomadegbe *et al.* (2000)	1.144760	(1.0369698-1.2637535)	
	Ryan *et al.* (2001)	1.157872	(1.0473412-1.2800682)	
	Hamajima *et al.* (2002)	1.167436	(1.0584582-1.2876343)	
	Hamajima *et al.* (2002)	1.160302	(1.0494526-1.2828590)	
	Hamajima *et al.* (2002)	1.161420	(1.0507138-1.2837900)	
	Hiraki *et al.* (2003)	1.159823	(1.0487431-1.2826670)	
	Huang *et al.* (2003)	1.163557	(1.0529857-1.2857395)	
	Hishida *et al.* (2004)	1.142271	(1.0333188-1.2627111)	
	Li(a) *et al.* (2004)	1.143728	(1.0342451-1.2647996)	
	Li(b) *et al.* (2004)	1.145781	(1.0329837-1.2708955)	
	Niwa(a) *et al.* (2004)	1.144593	(1.0318558-1.2696481)	
	Hu *et al.* (2005)	1.146866	(1.0365896-1.2688736)	
	Niwa(b) *et al.* (2005)	1.159329	(1.0482337-1.2821976)	
	Pfeifer *et al.* (2005)	1.177307	(1.0732897-1.2914060)	
	Choi *et al.* (2006)	1.148775	(1.0391396-1.2699768)	

	Ge *et al.* (2006)	1.148268	(1.0367036-1.2718384)	
	Ge *et al.* (2006)	1.156936	(1.0446044-1.2813469)	
	Zheng *et al.* (2006)	1.152702	(1.0396528-1.2780446)	
	Chen *et al.* (2008)	1.148139	(1.0385279-1.2693180)	
	Li(c) *et al.* (2008)	1.155061	(1.0430828-1.2790595)	
	Zheng *et al.* (2008)	1.144066	(1.0320406-1.2682507)	
	Deo Feo *et al.* (2009)	1.157241	(1.0464940-1.2797089)	
	Kang *et al.* (2009)	1.125273	(1.0262575-1.2338406)	
	Misra *et al.* (2009)	1.164561	(1.0541711-1.2865114)	
	Lee *et al.* (2010)	1.153768	(1.0426797-1.2766911)	
	Shirai *et al.* (2010)	1.141204	(1.0315313-1.2625375)	
	Arfaoui *et al.* (2010)	1.157273	(1.0450821-1.2815067)	
	Mittal *et al.* (2011)	1.150345	(1.0394986-1.2730113)	
	Craveiro *et al.* (2012)	1.155567	(1.0447082-1.2781895)	
	Sun *et al.* (2012)	1.163913	(1.0537320-1.2856148)	
	Umar *et al.* (2012)	1.144549	(1.0345836-1.2662033)	
	Zhou *et al.* (2012)	1.139914	(1.0316197-1.2595774)	
	Zhang *et al.* (2014)	1.166127	(1.0564353-1.2872078)	
	Wang *et al.* (2014)	1.142446	(1.0326366-1.2639317)	
	Feng *et al.* (2017)	1.148600	(1.0380493-1.2709239)	
	Combined	1.152058	(1.0435261-1.2718780)	
BB vs. BA+AA	Romain *et al.* (1999)	1.280292	(1.0402069-1.5757894)	Random
	Ahomadegbe *et al.* (2000)	1.271044	(1.0339199-1.5625511)	
	Ryan *et al.* (2001)	1.303375	(1.0669432-1.5921984)	
	Hamajima *et al.* (2002)	1.268349	(1.0288452-1.5636075)	
	Hamajima *et al.* (2002)	1.265541	(1.0260426-1.5609418)	
	Hamajima *et al.* (2002)	1.262319	(1.0234557-1.5569305)	
	Hiraki *et al.* (2003)	1.265281	(1.0254469-1.5612091)	
	Huang *et al.* (2003)	1.262941	(1.0222231-1.5603449)	
	Hishida *et al.* (2004)	1.256862	(1.0183030-1.5513074)	
	Li(a) *et al.* (2004)	1.295471	(1.0545678-1.5914063)	
	Li(b) *et al.* (2004)	1.288746	(1.0424550-1.5932254)	

Niwa(a) *et al.* (2004)	1.262680	(1.0157177-1.5696899)
Hu *et al.* (2005)	1.242432	(1.0097862-1.5286763)
Niwa(b) *et al.* (2005)	1.244930	(1.013824-1.5287175)
Pfeifer *et al.* (2005)	1.316514	(1.0750323-1.6122388)
Choi *et al.* (2006)	1.284109	(1.0445638-1.5785881)
Ge *et al.* (2006)	1.274597	(1.0314537-1.5750570)
Ge *et al.* (2006)	1.268475	(1.0252775-1.5693581)
Zheng *et al.* (2006)	1.268545	(1.0236333-1.5720526)
Chen *et al.* (2008)	1.287632	(1.0480676-1.5819544)
Li(c) *et al.* (2008)	1.279418	(1.0356594-1.5805490)
Zheng *et al.* (2008)	1.219571	(1.0345848-1.4376339)
Deo Feo *et al.* (2009)	1.255708	(1.0191657-1.5471506)
Kang *et al.* (2009)	1.259387	(1.0209192-1.5535556)
Misra *et al.* (2009)	1.267725	(1.0262104-1.5660799)
Lee *et al.* (2010)	1.256608	(1.0167701-1.5530203)
Shirai *et al.* (2010)	1.263744	(1.0211021-1.5640445)
Arfaoui *et al.* (2010)	1.274572	(1.0304883-1.5764707)
Mittal *et al.* (2011)	1.290407	(1.0535577-1.5805021)
Craveiro *et al.* (2012)	1.276300	(1.0352232-1.5735163)
Sun *et al.* (2012)	1.304202	(1.0631860-1.5998540)
Umar *et al.* (2012)	1.284534	(1.0419721-1.5835621)
Zhou *et al.* (2012)	1.252978	(1.0185923-1.5412984)
Zhang *et al.* (2014)	1.332216	(1.0945079-1.6215501)
Wang *et al.* (2014)	1.267262	(1.0263388-1.5647396)
Feng *et al.* (2017)	1.284721	(1.0423905-1.5833875)
Combined	1.273473	(1.0375487-1.5630421)

Supplementary table 4. *P* values of the Egger's test for *TP73 G4C14-A4T14* polymorphism.

Polymorphisms	Subgroup	Egger's test P > |t|
rs2273953	Overall	0.232
	Caucasian	0.533
	Asian	0.961
	Breast cancer	0.419
	Colorectal cancer	0.117
	SCCHN	0.765
	Cervical cancer	0.646
	Esophageal cancer	0.748
	Gastric cancer	0.739
	Lung cancer	0.202
	P-B	0.584
	H-B	0.322
	HWE(Y)	0.315
	HWE(N)	0.682

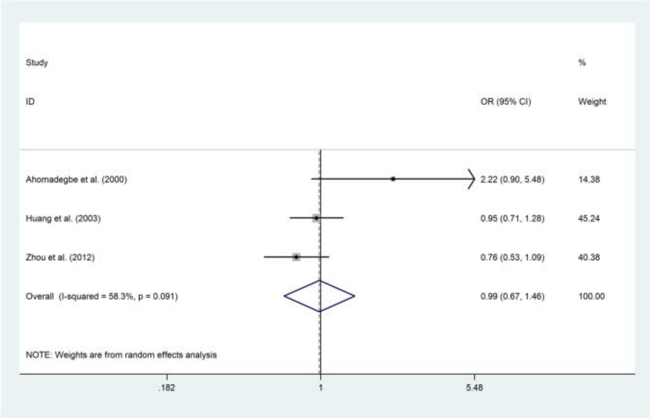

B VS A

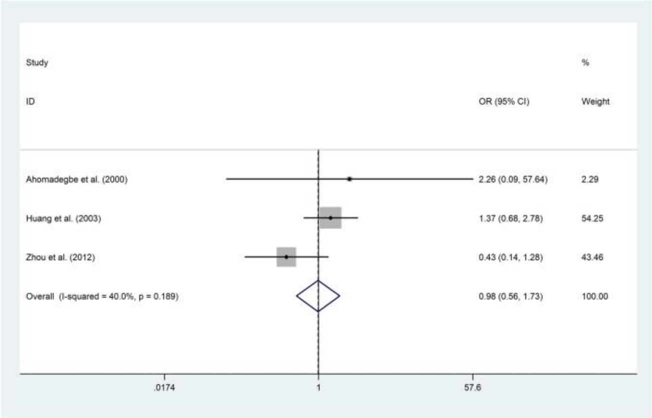

BB VS AA

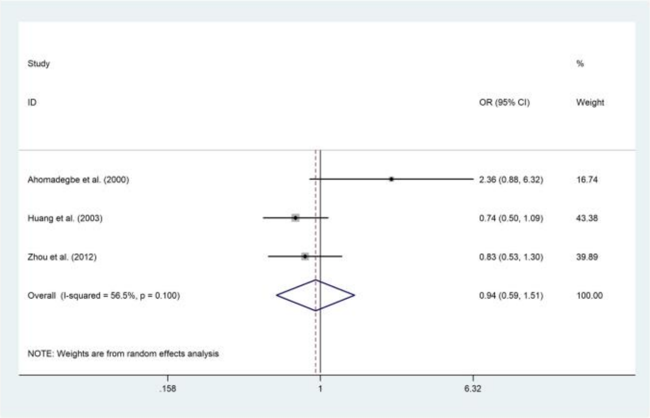

BA VS AA

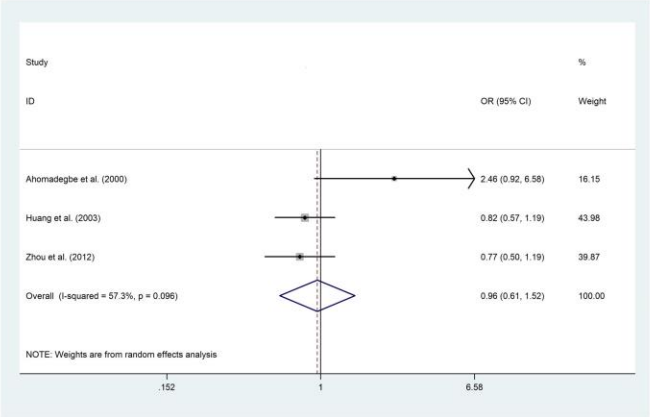

BB+BA VS AA

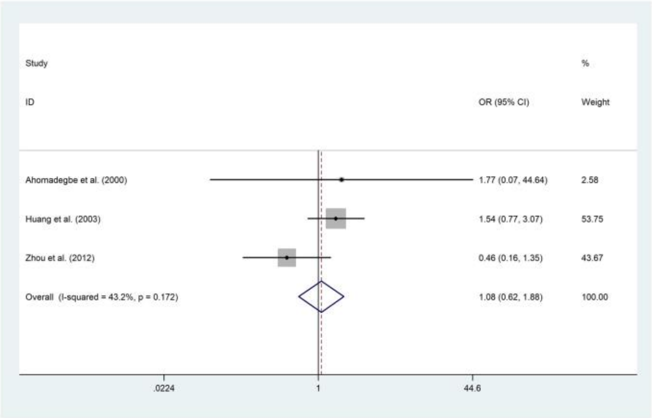

BB VS BA+AA

Fig.S1

Meta-analysis of the association between *TP73 G4C14-A4T14* polymorphism and breast cancer risk.

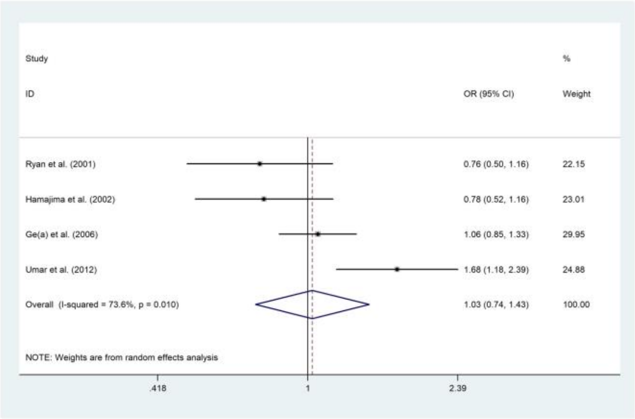

B VS A

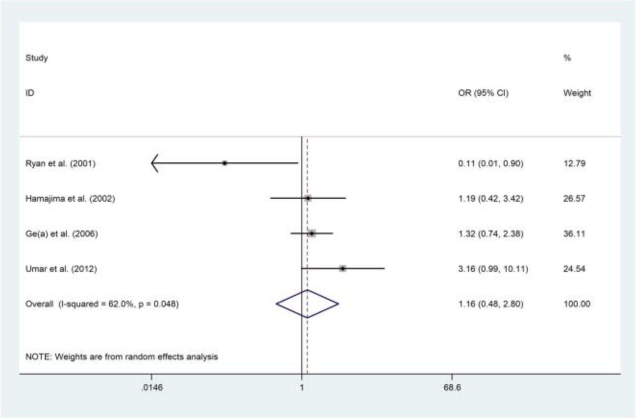

BB VS AA

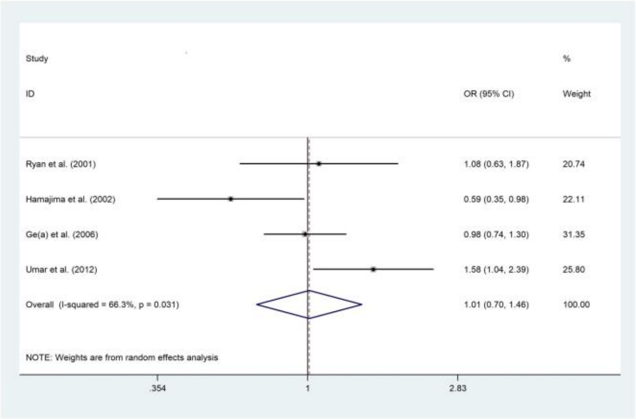

BA VS AA

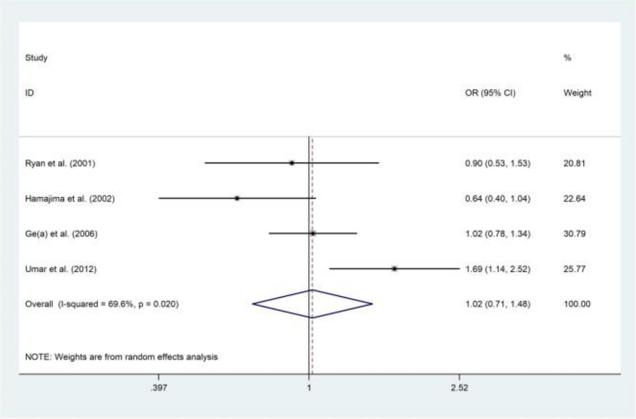

BB+BA VS AA

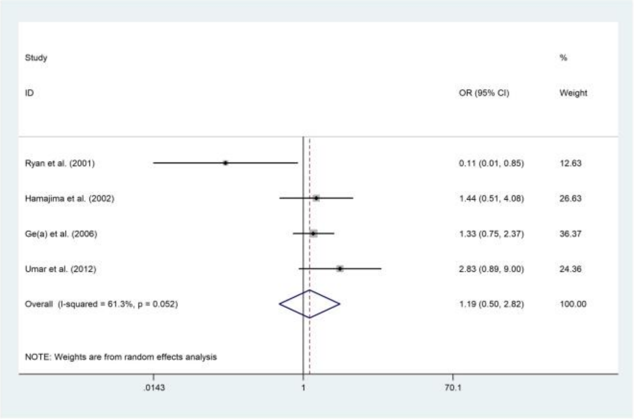

BB VS BA+AA

Fig.S2

Meta-analysis of the association between *TP73 G4C14-A4T14* polymorphism and esophageal cancer risk.

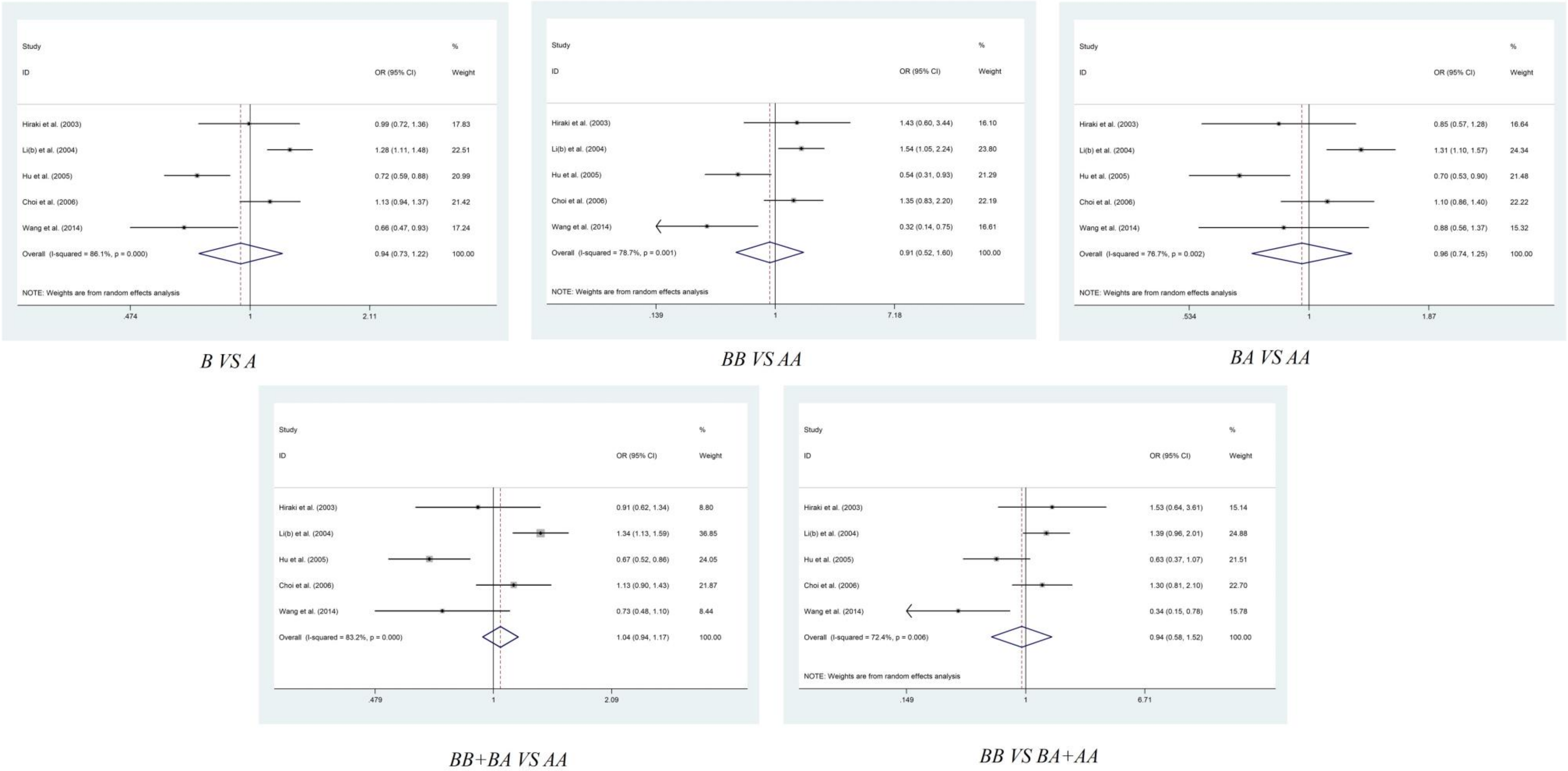

Fig.S3

Meta-analysis of the association between *TP73 G4C14-A4T14* polymorphism and lung cancer risk.

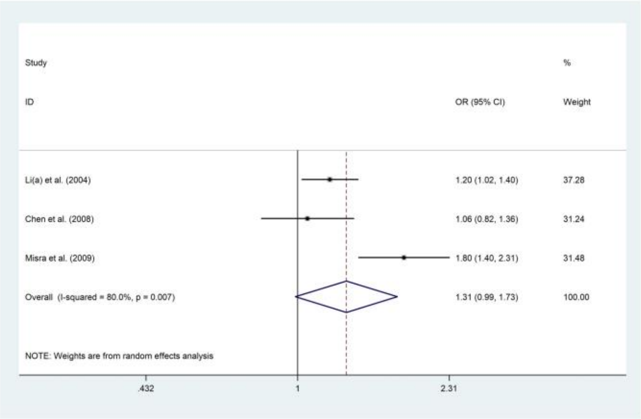

B VS A

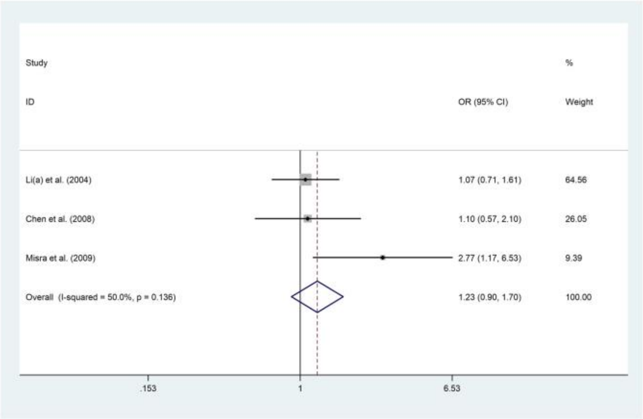

BB VS AA

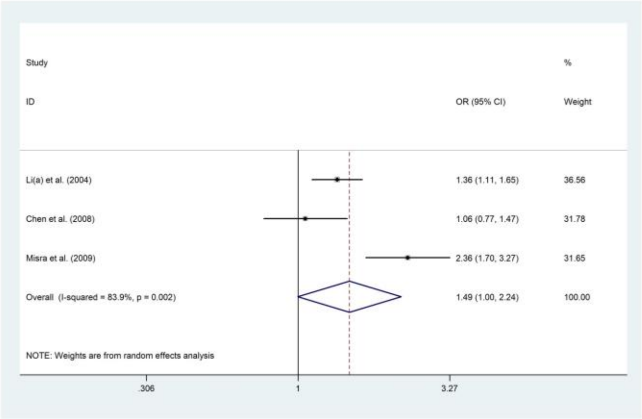

BA VS AA

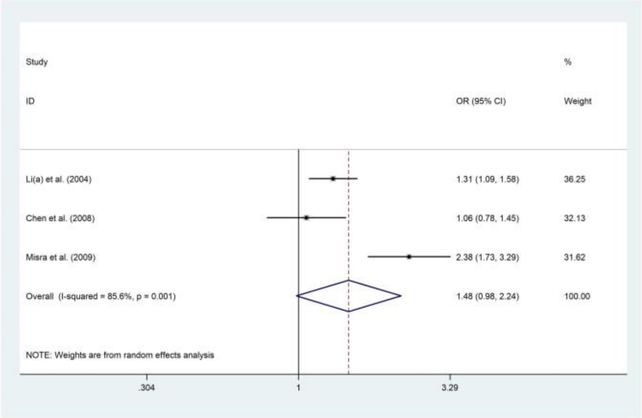

BB+BA VS AA

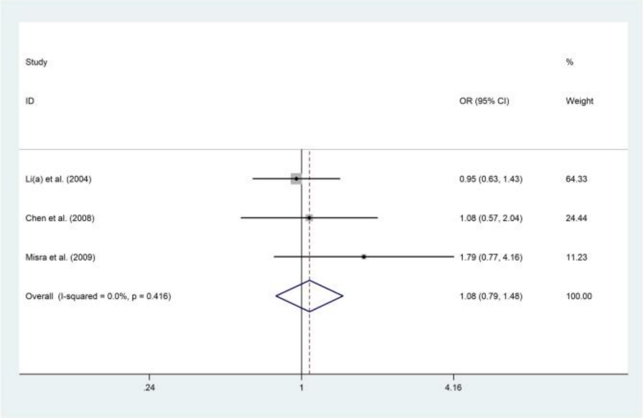

BB VS BA+AA

Fig.S4

Meta-analysis of the association between *TP73 G4C14-A4T14* polymorphism and squamous cell carcinoma of the head and neck cancer risk.

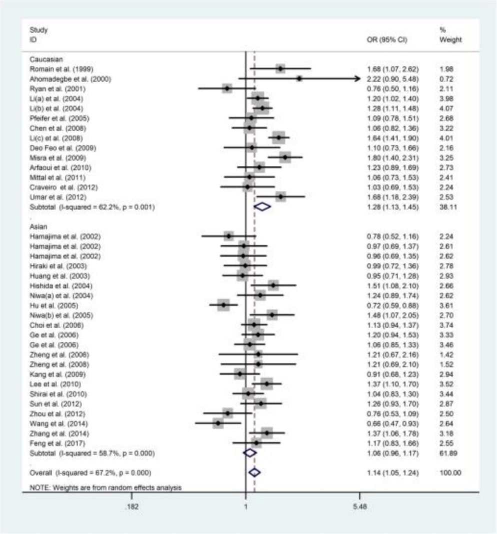

B VS A

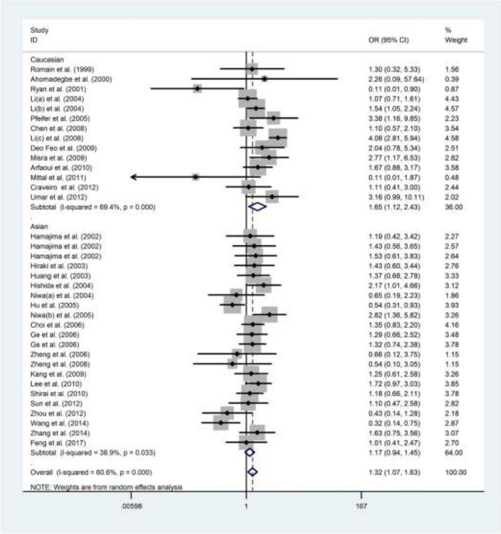

BB VS AA

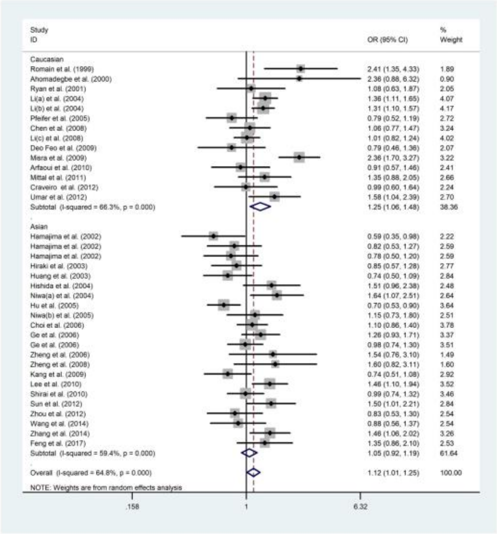

BA VS AA

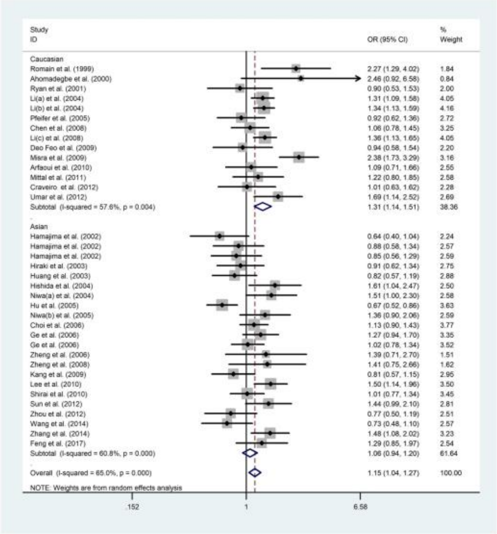

BB+BA VS AA

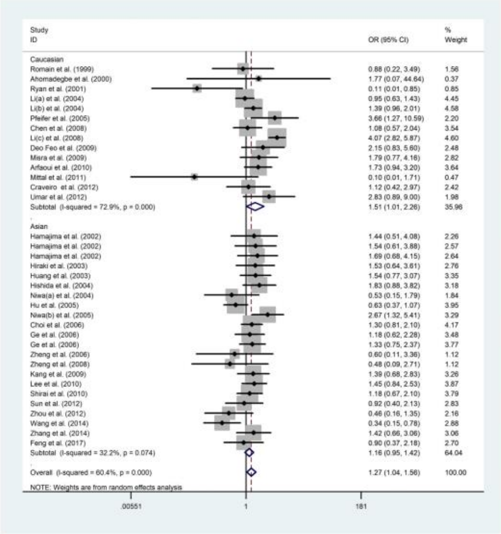

BB VS BA+AA

Fig.S5

Meta-analysis of the association between *TP73 G4C14-A4T14* polymorphism and cancer risks, stratification by ethnicity.

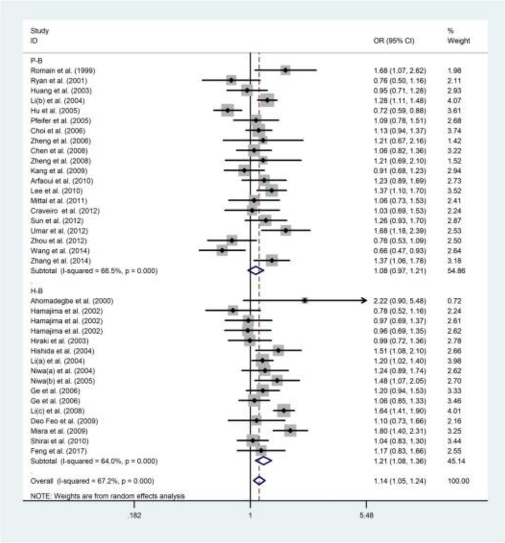

B VS A

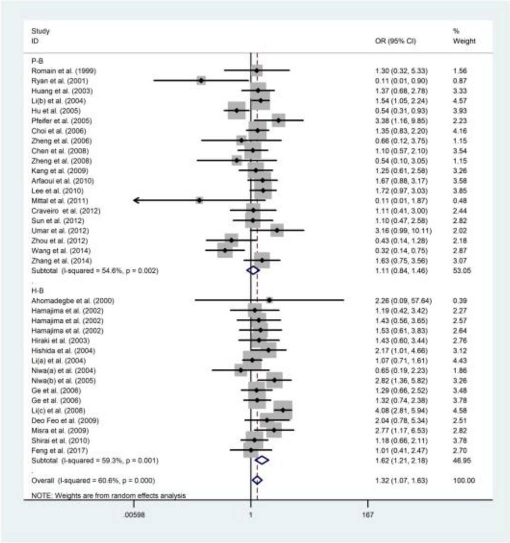

BB VS AA

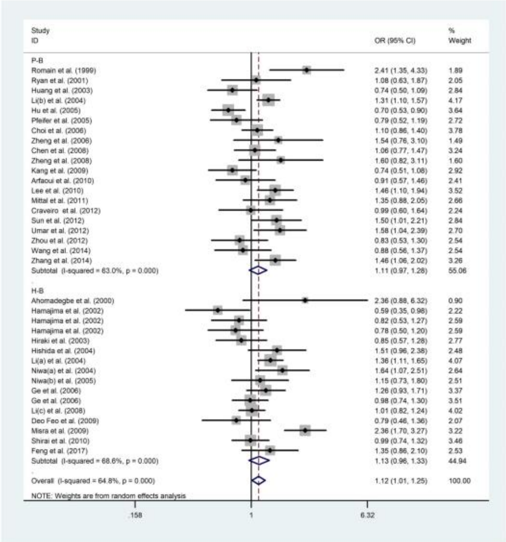

BA VS AA

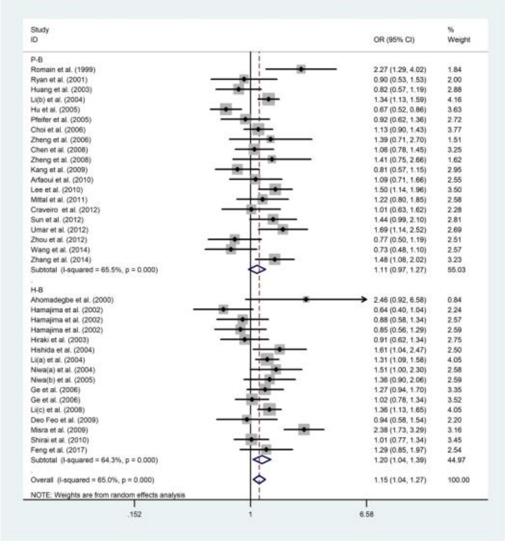

BB+BA VS AA

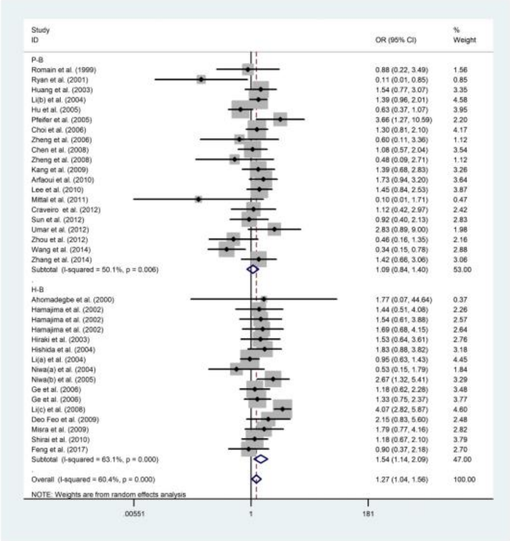

BB VS BA+AA

Fig.S6

Meta-analysis of the association between *TP73 G4C14-A4T14* polymorphism and cancer risks, stratification by source of control.

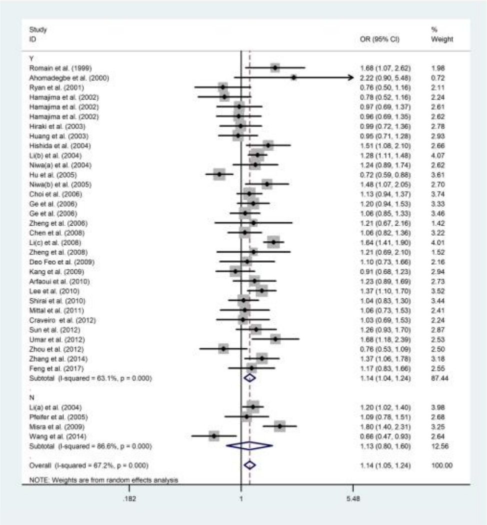

B VS A

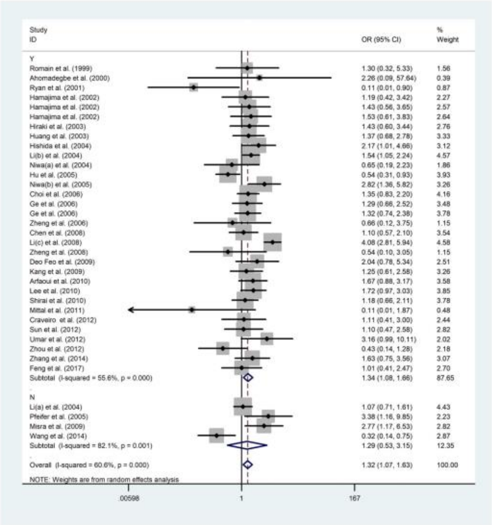

BB VS AA

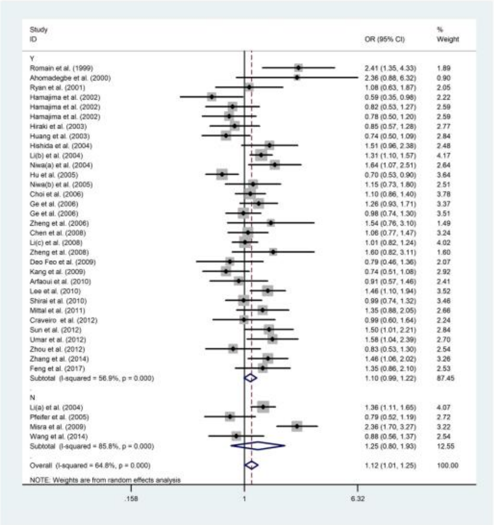

BA VS AA

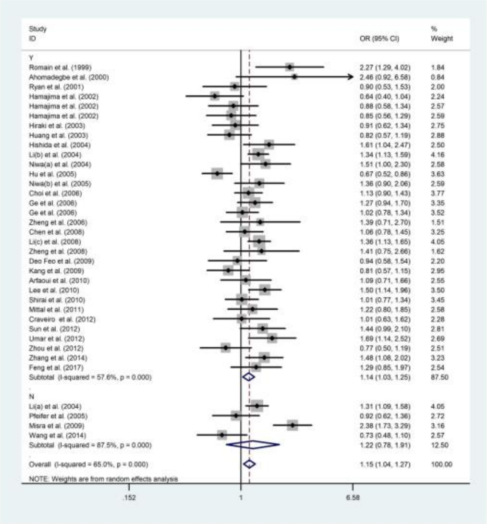

BB+BA VS AA

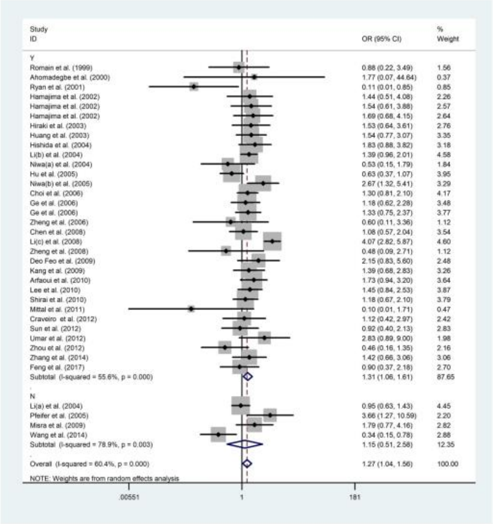

BB VS BA+AA

Fig.S7

Meta-analysis of the association between *TP73 G4C14-A4T14* polymorphism and cancer risks, stratification by whether conform to HWE.

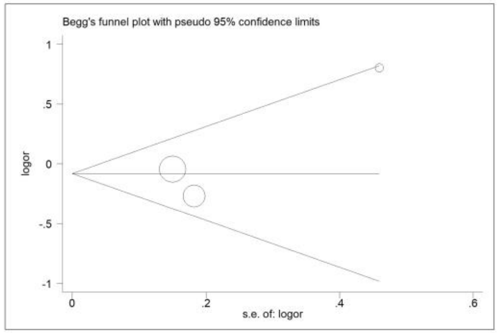

Breast Cancer

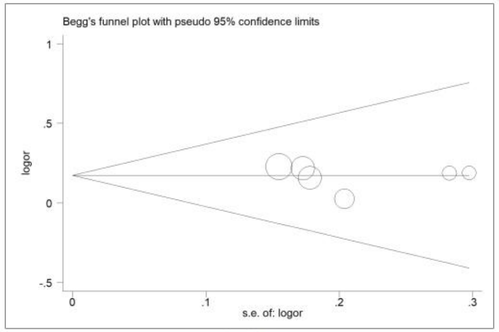

Cervical Cancer

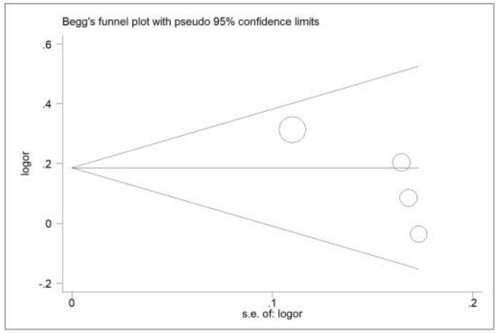

Colorectal Cancer

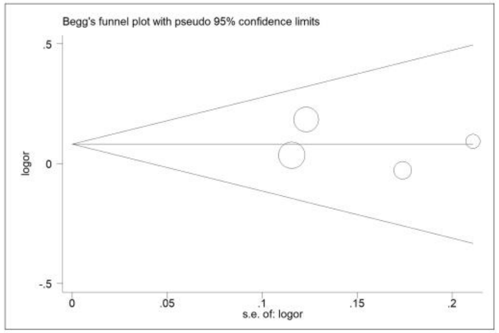

Gastric Cancer

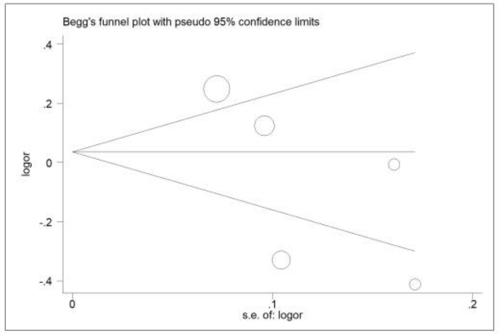

Lung Cancer

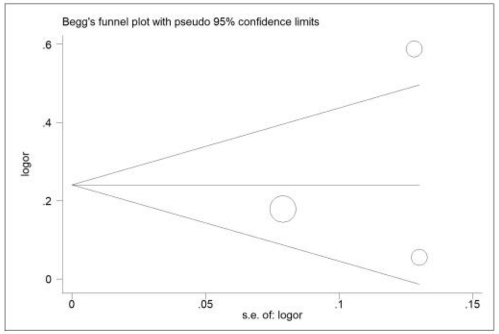

SCCHN

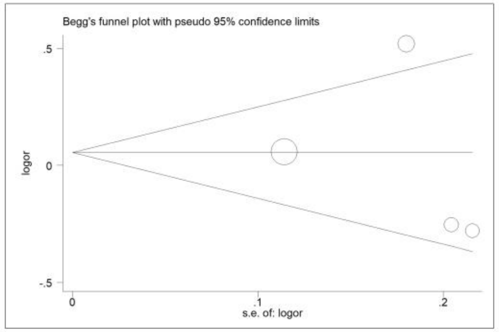

Esophageal Cancer

Fig.S8

Begg's funnel plot for publication bias test for TP73 G4C14-A4T14 polymorphism (B vs. A), stratification by cancer type. The x-axis is log (OR), and the y-axis is natural logarithm of OR. The horizontal line in the figure represents the overall estimated log (OR). The two diagonal lines indicate the pseudo 95% confidence limits of the effect estimate.

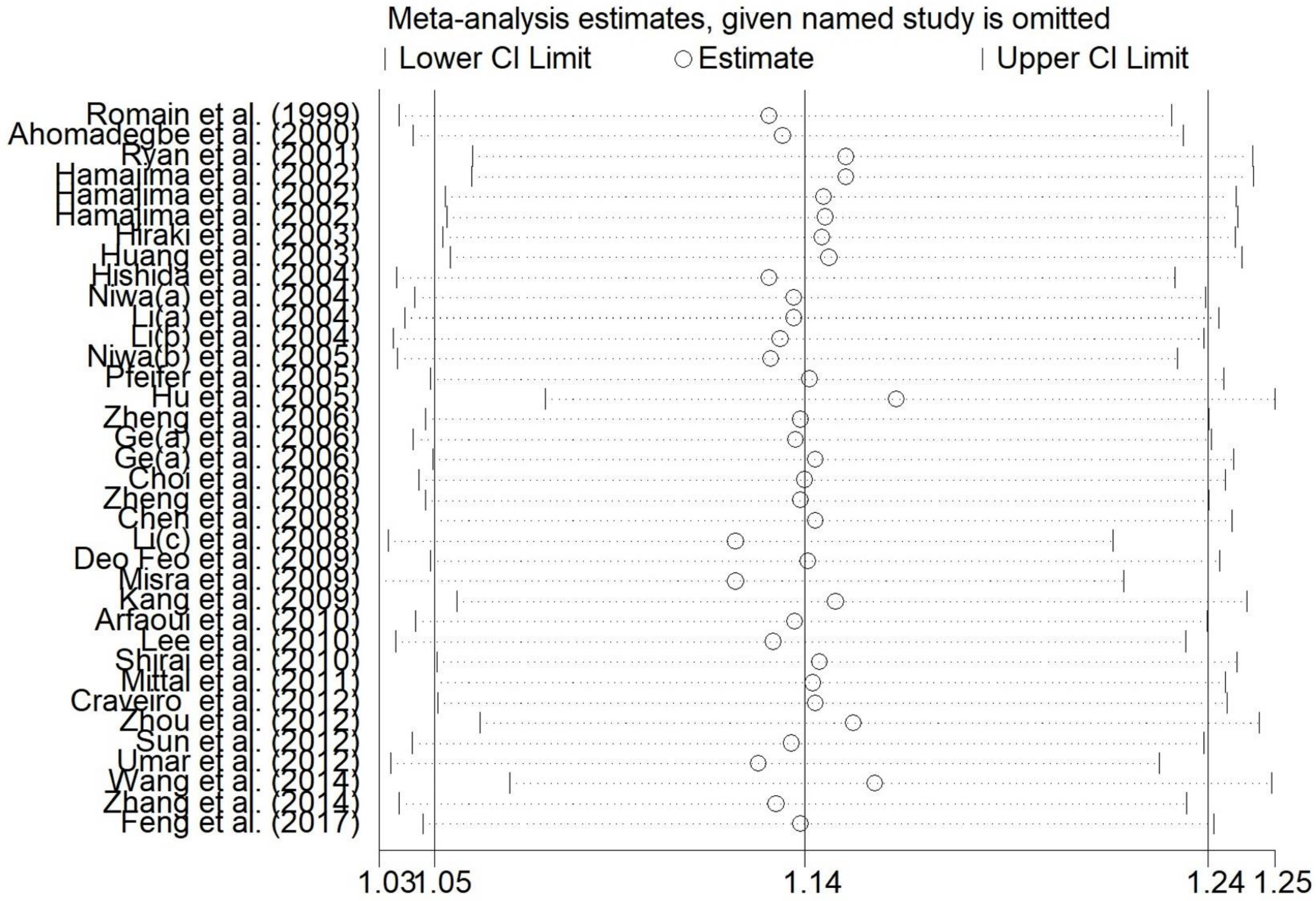

Fig.6

Fig. S9 Sensitivity Analysis of Overall ORs Co-Efficients for *TP73 G4C14-A4T14* polymorphism (GC vs. AT). Results were calculated by omitting each study in turn. The two ends of the dotted lines represent the 95% CIs.

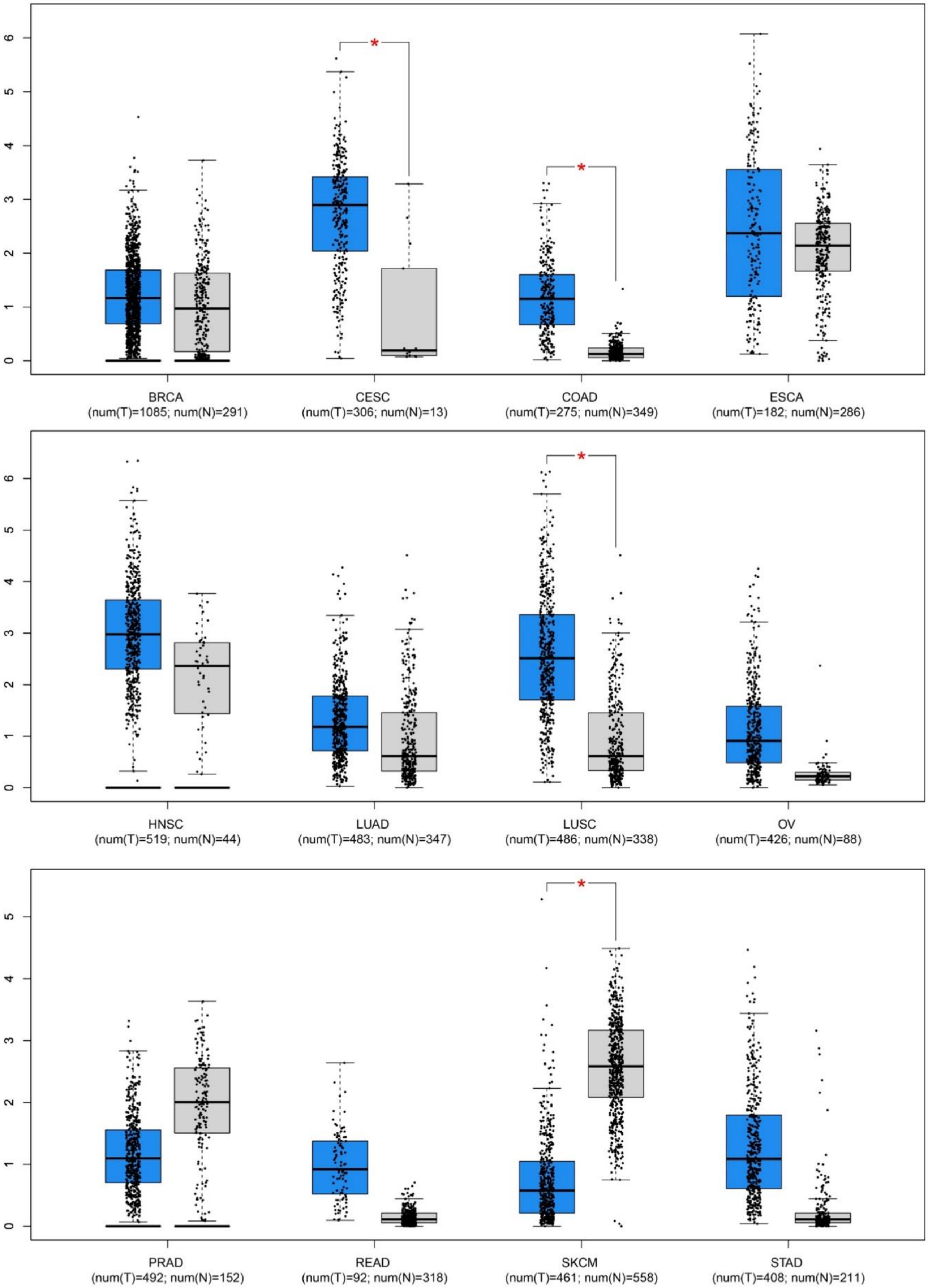

Fig. S10 In-silico analysis the relative expression of p73 in cancer tissue and normal tissue using TCGA database. BRCA: Breast invasive carcinoma; CESC: Cervical squamous cell carcinoma and endocervical adenocarcinoma; COAD: Colon adenocarcinoma; ESCA: Esophageal carcinoma; HNSC: Head and Neck squamous cell carcinoma; LUAD: Lung adenocarcinoma; LUSC: Lung squamous cell carcinoma; OV: Ovarian serous; PRAD: Prostate adenocarcinoma; READ: Rectum adenocarcinoma; SKCM: Skin Cutaneous Melanoma; STAD: Stomach adenocarcinoma.
